# Investigating the microbial ecology of coastal hotspots of marine nitrogen fixation in the western North Atlantic

**DOI:** 10.1038/s41598-021-84969-1

**Published:** 2021-03-09

**Authors:** Seaver Wang, Weiyi Tang, Erwan Delage, Scott Gifford, Hannah Whitby, Aridane G. González, Damien Eveillard, Hélène Planquette, Nicolas Cassar

**Affiliations:** 1 Division of Earth and Ocean Sciences, Duke University, Grainger Environment Hall, 9 Circuit Drive, Box 90328, Durham, NC 27708 USA; 2 Department of Geosciences, Princeton University, Princeton, NJ USA; 3 LS2N, UMR 6004, CNRS, Université de Nantes, 44000 Nantes, France; 4 Department of Marine Sciences, The University of North Carolina at Chapel Hill, Chapel Hill, NC USA; 5 Department of Earth, Ocean, and Ecological Sciences, School of Environmental Sciences, University of Liverpool, Liverpool, UK; 6 Instituto de Oceanografía y Cambio Global (IOCAG), Universidad de Las Palmas de Gran Canaria, ULPGC, Las Palmas, Spain; 7 Laboratoire des Sciences de l’Environnement Marin (LEMAR), Institut Universitaire Européen de la Mer (IUEM), Technopôle Brest-Iroise, 13 Plouzané, 29280 Locmaria-Plouzané, France

**Keywords:** Marine biology, Element cycles

## Abstract

Variation in the microbial cycling of nutrients and carbon in the ocean is an emergent property of complex planktonic communities. While recent findings have considerably expanded our understanding of the diversity and distribution of nitrogen (N_2_) fixing marine diazotrophs, knowledge gaps remain regarding ecological interactions between diazotrophs and other community members. Using quantitative 16S and 18S V4 rDNA amplicon sequencing, we surveyed eukaryotic and prokaryotic microbial communities from samples collected in August 2016 and 2017 across the Western North Atlantic. Leveraging and significantly expanding an earlier published 2015 molecular dataset, we examined microbial community structure and ecological co-occurrence relationships associated with intense hotspots of N_2_ fixation previously reported at sites off the Southern New England Shelf and Mid-Atlantic Bight. Overall, we observed a negative relationship between eukaryotic diversity and both N_2_ fixation and net community production (NCP). Maximum N_2_ fixation rates occurred at sites with high abundances of mixotrophic stramenopiles, notably *Chrysophyceae*. Network analysis revealed such stramenopiles to be keystone taxa alongside the haptophyte diazotroph host *Braarudosphaera bigelowii* and chlorophytes. Our findings highlight an intriguing relationship between marine stramenopiles and high N_2_ fixation coastal sites.

## Introduction

Marine phytoplankton mediate significant fluxes of carbon, oxygen, nitrogen, and other elements between organic, atmospheric, and oceanic pools. Nitrogen (N_2_) fixation is a critical biogeochemical process in which specialized marine prokaryotes provide the surface planktonic community with a supply of new nitrogen obtained from the atmosphere. N_2_ fixation supports marine primary production and can increase the sequestration of biological carbon in the deep ocean via sinking plankton biomass^1^. As a result, marine N_2_ fixation is an important process influencing the ocean carbon cycle, with implications for marine carbon sink strength and global climate^1–3^.

Marine N_2_ fixation and the diazotrophic microbes that perform this function are strongly influenced by nutrient availability, ecosystem conditions, and the ecology and structure of the surface microbial community^4–6^. Consequently, microbial oceanographers are devoting considerable effort to studying the links between N_2_ fixation, the physical and chemical environment, and community structure. While studies of bottom-up controls on N_2_ fixation are well underway^5,7–9^, the complex ecological relationships governing diazotrophs remain only partially explored. For example, there is increasing field evidence regarding the significance of top-down factors in controlling diazotroph populations^10–12^, potentially influencing the global distribution of diazotrophs^13^. Our evolving understanding of such ecological dynamics stems from discoveries over the last decade that have significantly altered the scientific knowledge behind marine N_2_ fixation. Researchers have discovered a number of additional marine diazotrophs, including UCYN-A—a cyanobacterial endosymbiont of the haptophyte phytoplankton *Braarudosphaera bigelowii*^14,15^—and numerous groups of non-cyanobacterial diazotrophs (NCDs), including members of Gammaproteobacteria and Planctomycetes^10,16–18^. Other key discoveries include the finding that active marine N_2_ fixation can occur in the presence of nitrate or ammonium^19–21^, that coastal rates of N_2_ fixation are much higher than previously thought^22,23^, and that N_2_ fixation can be significant even in cold high-latitude regions^20,24,25^, as reviewed in Zehr and Capone, 2020^5^.

Such revelations also make it clear that the surrounding microbial community likely exerts ecological influences over diazotrophs in addition to the physical environment and nutrient regime. Colony-forming or host-associated N_2_ fixing bacteria may interact with other marine microbes inhabiting colony or host cell surfaces^26–28^. Community interactions might also provide diazotrophs with sources of organic-rich particles and aggregates^29^, and recycled essential nutrients such as phosphorus^30^. Grazers^11,31^, viruses^32^, and parasites represent other ways in which ecological dynamics might regulate N_2_ fixing organisms and their hosts via predation upon the host algal cells. Finally, the marine environment itself can be altered by local microbial ecology, such as the transmissivity of the water column to light^33^ or the presence of inhibitory compounds produced by competitors, particularly in more restricted coastal waters or at very high cell densities^34^. Marine microbial community structure thus potentially moderates N_2_ fixation activity^35^. Hotspots of N_2_ fixation might be considered an emergent property of the whole marine microbial community. Such possibilities add considerable value to uncovering ecological conditions or relationships between marine diazotrophs and other taxa within the surface ocean community. While conclusively linking N_2_ fixation to microbial abundances and diversity requires understanding underlying ecological mechanisms, we remain at a stage where inferences from the field are necessary to highlight patterns of interest for focused study.

From August 2015 to August 2017, a successive series of research expeditions in the western North Atlantic aboard the *R/V Atlantic Explorer* sought to pair underway measurements of N_2_ fixation with sampling of diazotroph and marine microbial community structure, biogeochemical properties, and a survey of net community production rates. Wang et al., 2018 described a marine microbial community dominated by *Aureococcus anophagefferens* associated with a coastal site in the Mid-Atlantic Bight exhibiting elevated net community production rates^36^. Using a new method for high-resolution underway observations of N_2_ fixation, Tang et al., 2019 identified these productive coastal waters of the Mid-Atlantic Bight and Southern New England Shelf also to be hotspots of elevated N_2_ fixation dominated by the cyanobacterial diazotroph UCYN-A, with extremely high rates up to 167 µmol N m^−3^ day^−1^ measured off the New Jersey coast^22^. Tang et al. 2020 analyzed macronutrient and micronutrient concentrations within coastal ecosystems with high rates of N_2_ fixation. They discovered these environments to be relatively replete in iron and nitrate, identifying phosphorus and temperature as key factors governing N_2_ fixation activity and diazotrophic community structure, respectively^37^. In the same study, a *nifH* molecular survey identified UCYN-A as dominant in cold, subpolar coastal waters with diazotroph populations shifting towards *Trichodesmium* in warmer, oligotrophic waters offshore.

In this study, we investigated the microbial diversity and ecological relationships underlying the published extensive regional survey of marine N_2_ fixation rates in summer reported in Tang et al., 2019 and Tang et al., 2020. Specifically, through statistical inferences, we attempted to assess potential competitive interactions associated with previously-identified coastal hotspots of N_2_ fixation, refining our understanding of the ecological niche occupied by diazotrophic plankton in this ecosystem. We hypothesize that given previous findings of record UCYN-A abundances, network-based statistical analysis of co-occurrence patterns will reveal that UCYN-A’s host *B. bigelowii* plays a disproportionately crucial ecological role within this productive coastal community, potentially reflecting its role in transferring bioavailable N into the microbial environment.

## Results and discussion

### Microbial ecology: marine microbial community composition and spatial and abundance patterns

The earlier discovery that UCYN-A is most likely responsible for observed coastal peaks in N_2_ fixation in the Mid-Atlantic Bight and Southern New England Shelf^22^ is intriguing given that UCYN-A is presumed to be an obligate endosymbiont of its host *Braarudosphaera bigelowii*. UCYN-A’s record abundances (up to 4 × 10^7^ *nifH* copies L^−1^)^37^ raise the question of what ecological conditions permit its host organism to be competitively successful and whether locally elevated N_2_ fixation rates could be partially enabled by the success of the host phytoplankton in addition to the favorability of a niche for diazotrophic N_2_ fixation.

**Figure 1:**
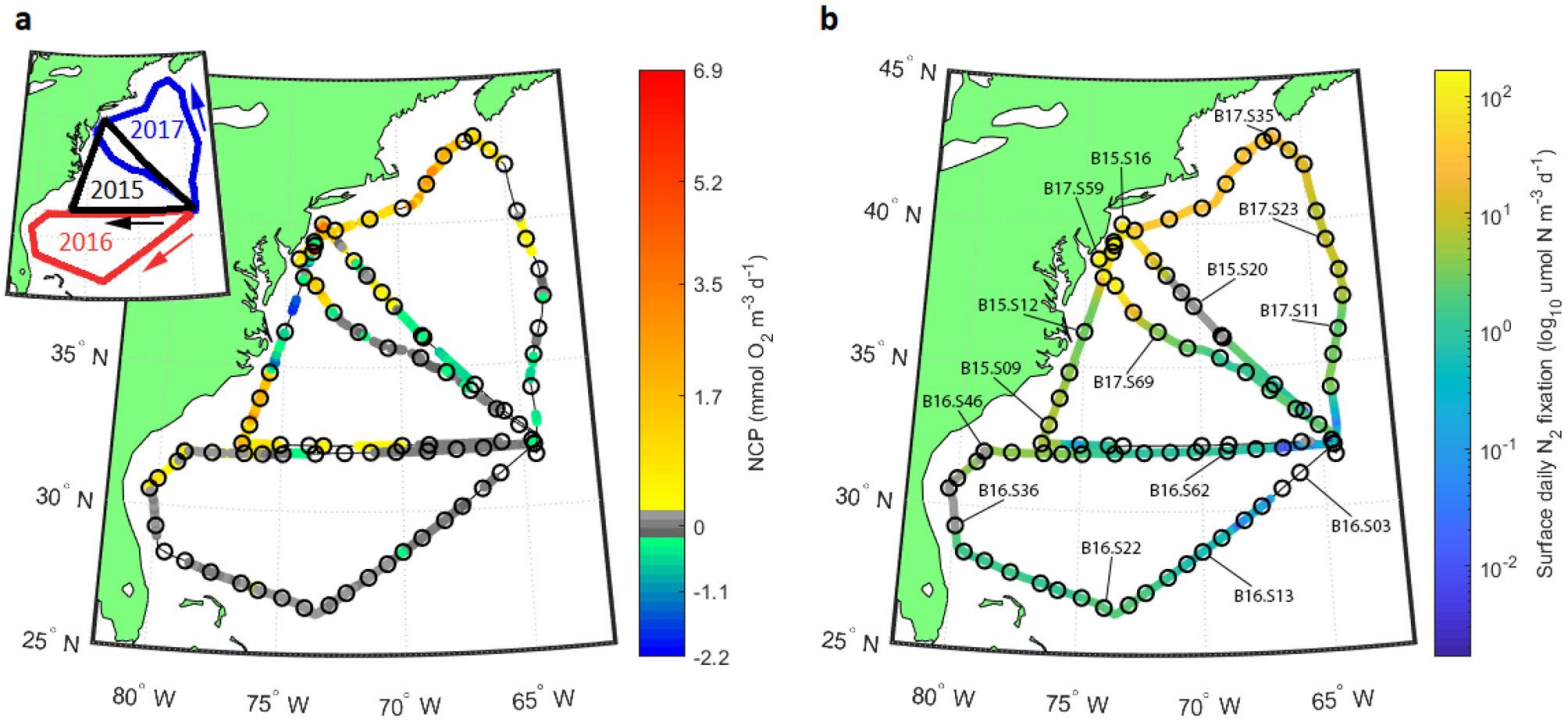
NCP and N_2_ fixation rates. (**a)** Map of O_2_/Ar-based volumetric NCP rates measured via EIMS across the Western North Atlantic in July–August 2015^36^, 2016, and 2017^37^. Positive rates are represented by the warm portion of the color scale, while negative rates are indicated using a cool color scale. Balanced NCP rates are shown in greyscale tones. Sites of molecular sampling are displayed by open circles. (**b)** Map of surface daily N_2_ fixation rates measured using the FARACAS method^22,37^. Sites of molecular sampling are displayed by open circles, with selected sites labeled. Greyscale points indicate sites at which N_2_ fixation rates were below the limit of detection for the FARACAS method (0.19 µmol N m^-3^ day^-1^). Inset plot illustrates cruise tracks and directions of travel, color-coded for each year of sampling. Plots created using Matlab 2018b (https://www.mathworks.com/products/matlab.html).

**Figure 2:**
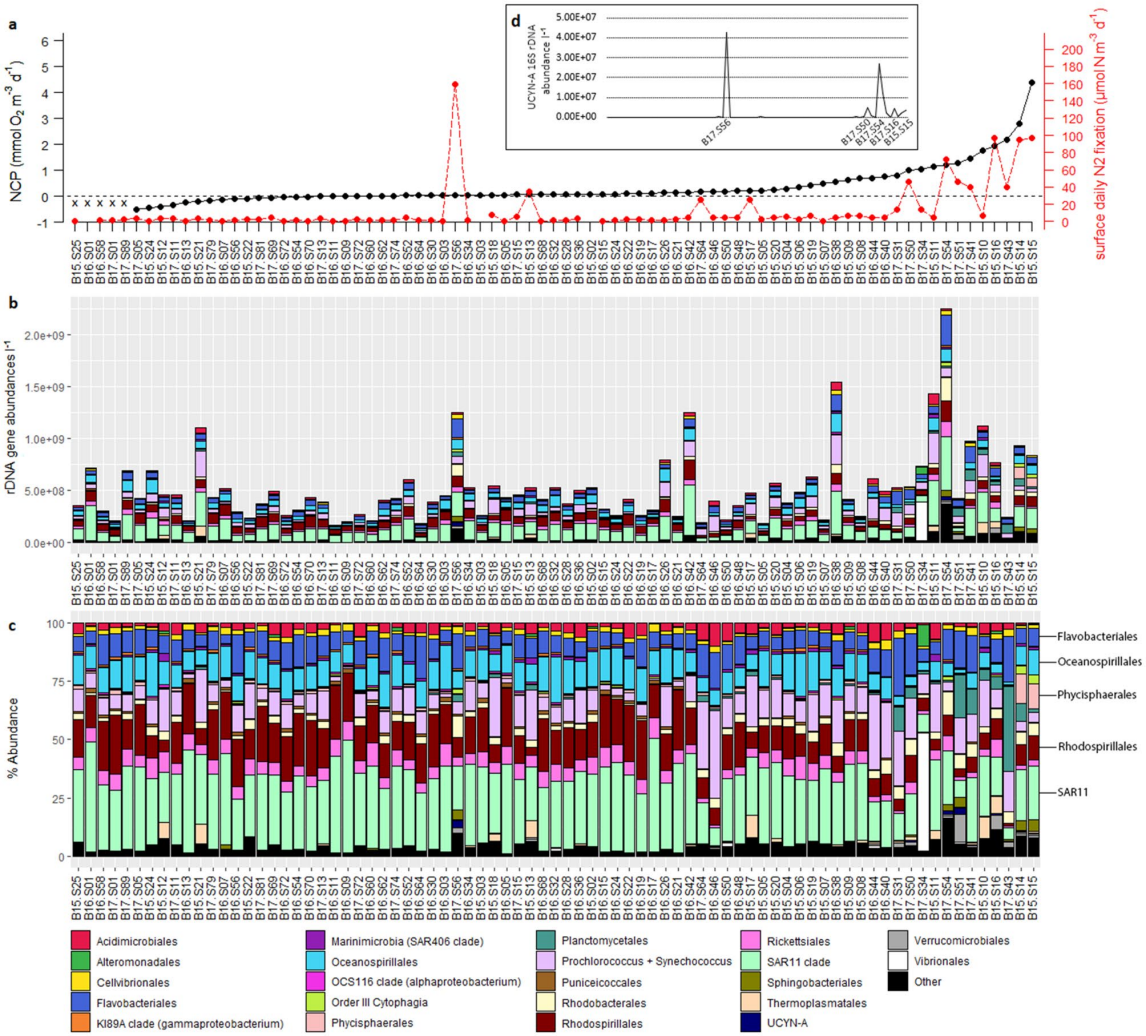
Prokaryotic community structure. (**a)** N_2_ fixation rates (red) and NCP rates (black) measured at each sampling site^22,36,37^, with sites ranked from left-to-right in order of increasing NCP. Sites at which NCP measurements were not collected are grouped on the far left and denoted with x-marks. Taxa are organized in alphabetical order from top to bottom, following the order outlined in the legend. (**b)** Bar plot of 16S prokaryotic taxonomy represented at the order level using quantitative rDNA gene abundances l^-1^. (**c)** Bar plot of 16S prokaryotic taxonomy at the order level using relative abundances. (**d)** UCYN-A1 16S rDNA abundance across sampling sites, with sites ranked from left-to-right in order of increasing NCP and selected sites labeled. Figure created in R 3.5.2 (https://www.r-project.org/).

In our molecular survey across the western North Atlantic (Fig. 1), marine prokaryotic 16S rDNA samples typically displayed high abundances of SAR11 and *Prochlorococcus* as well as appreciable fractions of Bacterioplankton belonging to the SAR86 clade (*Oceanospirillales)*, *Flavobacteriales*, and *Rhodospirillales*. A noticeable shift in community composition towards greater fractions of *Planctomycetes*, *Phycisphaerales*, and *Sphingobacteriales* was observed for stations with high N_2_ fixation rates and net community production (NCP) rates (Fig. 2).

The highest N_2_ fixation and NCP rates corresponded to samples collected in 2015 and 2017 near the New Jersey coast in the Mid-Atlantic Bight (MAB) and off the Southern New England Shelf (Fig. 1). Over the region studied, previous work^22,36,37^ reported surface daily N_2_ fixation rates ranging between undetectable values (< 0.19 μmol N m^−3^ day^−1^, see Cassar et al., 2018^38^) and 167 µmol N m^−3^ day^−1^ (mean ± sd: 3 ± 35.1 µmol N m^−3^ day^−1^) and volumetric NCP rates ranging between − 2.2 and 6.9 mmol O_2_ m^−3^ day^−1^ (0.4 ± 0.9 mmol O_2_ m^−3^ day^−1^).

**Figure 3:**
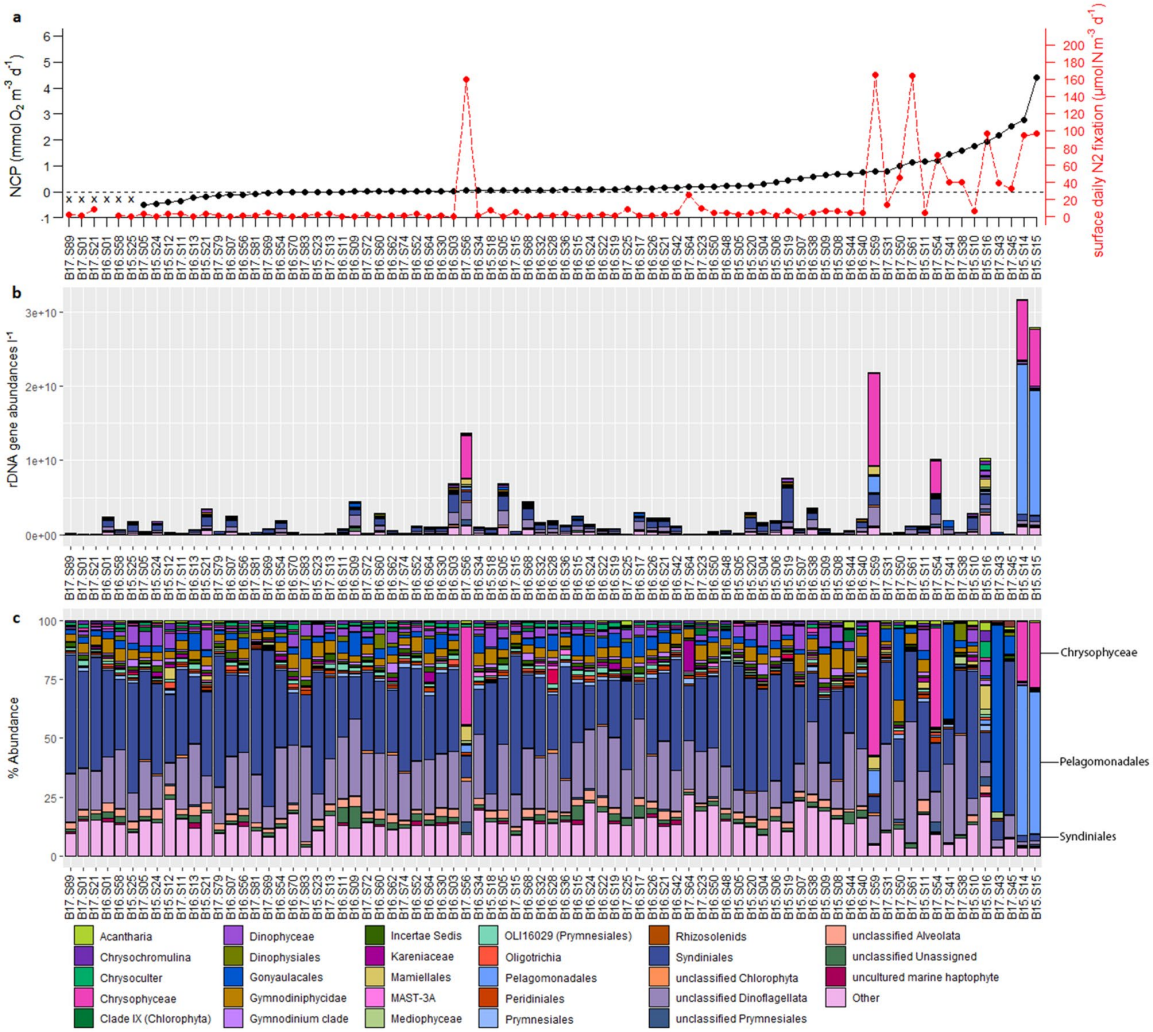
Eukaryotic community structure. (**a)** N_2_ fixation rates (red) and NCP rates (black) measured at each sampling site^22,36,37^, with sites ranked from left-to-right in order of increasing NCP. Sites at which NCP measurements were not collected are grouped on the far left and denoted with x-marks. Taxa are organized in alphabetical order from top to bottom, following the order outlined in the legend. (**b)** Bar plot of 18S eukaryotic taxonomy represented at the 5th taxonomic rank using quantitative rDNA gene abundances l^-1^. (**c)** Bar plot of 18S eukaryotic taxonomy at the 5th taxonomic rank using relative abundances. Note that *Aureococcus anophagefferens* fall within the order Pelagomonadales, and make up the overwhelming majority of pelagophytes in the sample set. Figure created in R 3.5.2 (https://www.r-project.org/).

Generally, eukaryotic 18S rDNA samples were dominated by dinoflagellate OTUs (Fig. 3), particularly *Syndiniales*—endoparasitic alveolates infecting a wide range of organisms from ciliates to cercozoa, zooplankton, and fish eggs^39–41^. The life cycle of *Syndiniales* also involves a free-living stage in which large numbers of *Syndiniales* are dispersed as dinospores, perhaps partially explaining their high abundances in many amplicon studies^39^. Eukaryotic dinoflagellates belonging to *Gonyaulacales*, *Gymnodiniphycidae*, and *Dinophyceae* appeared at around 5–10% relative abundances across most samples.

**Figure 4:**
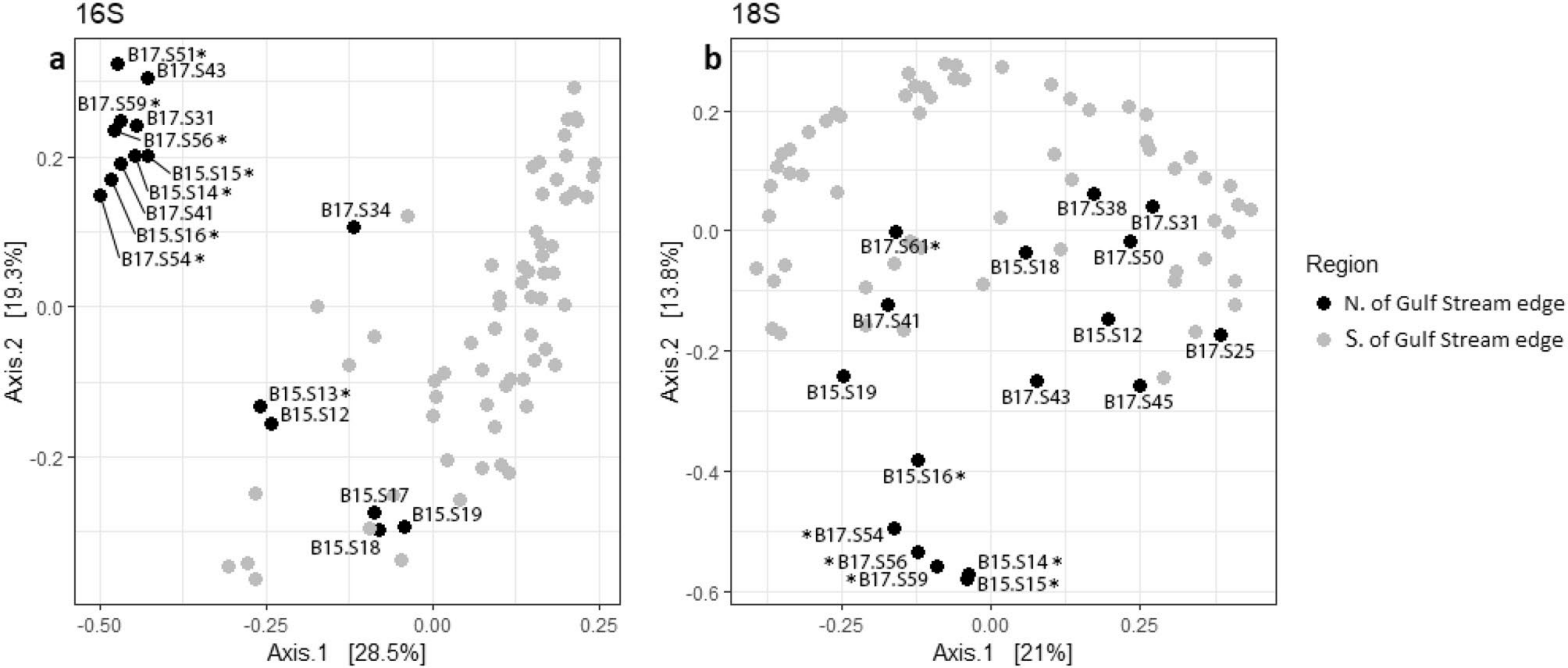
Principal Coordinates Analysis (PCoA) of (**a)** prokaryotic 16S and (**b)** eukaryotic 18S samples, ordinated using weighted Bray–Curtis dissimilarity. Samples are shaded based on whether they were collected north or south of the northern wall of the Gulf Stream in that year as determined from monthly EN4 temperature data^42^. Samples denoted with asterisks originate from the Mid-Atlantic Bight. Figure created in R 3.5.2 (https://www.r-project.org/).

Samples from the MAB and New England shelf exhibiting high N_2_ fixation and NCP rates in 2015 and 2017 were dominated by *Chrysophyceae* and the pelagophyte alga *Aureococcus anophagefferens*. Strikingly, of the seven stations with the highest N_2_ fixation rates, five showed extremely elevated *Chrysophyceae* abundances (25–55% of 18S rDNA abundances). Several of these samples also displayed elevated abundances of the chlorophyte *Mamiellales.* These samples from the MAB in August 2015 and 2017 clustered closely together during PCoA analysis, suggesting consistency in broad community structure between years (Fig. 4). Analysis of variance testing via the adonis function (999 permutations) in the “vegan” package revealed that the differences in community composition between 18 and 16S samples collected north of the Gulf Stream versus south of the Gulf Stream were statistically significant (p < 0.001).

Although we observed high N_2_ fixation, high NCP, and high abundances of *Chrysophyceae* in the MAB in both August 2015 and 2017, we note that *Aureococcus anophagefferens* did not bloom here to the same extent in 2017 as in 2015^36^. In 2017, *Aureococcus* occurred at 0.7–10.1% of the eukaryotic community within the MAB, with an estimated 7.2 × 10^7^ to 2.2 × 10^9^ 18S rDNA gene sequences l^−1^, contrasted with 2015 sampling (1.8 × 10^8^ to 2.0 × 10^10^ rDNA genes l^−1^). The absence of a similarly dramatic 2017 *Aureococcus* bloom in the MAB suggests that such events may not recur annually, or that blooms may be subject to variable timing and magnitude. Existing literature reporting upon coastal monitoring of *Aureococcus* indeed demonstrate that *Aureococcus* abundances may fluctuate by an order of magnitude between months during the growing season and that the seasonal biomass peak may precede the start of August by several weeks^43^.

### Broad patterns: community diversity vs. N_2_ fixation and NCP

**Figure 5:**
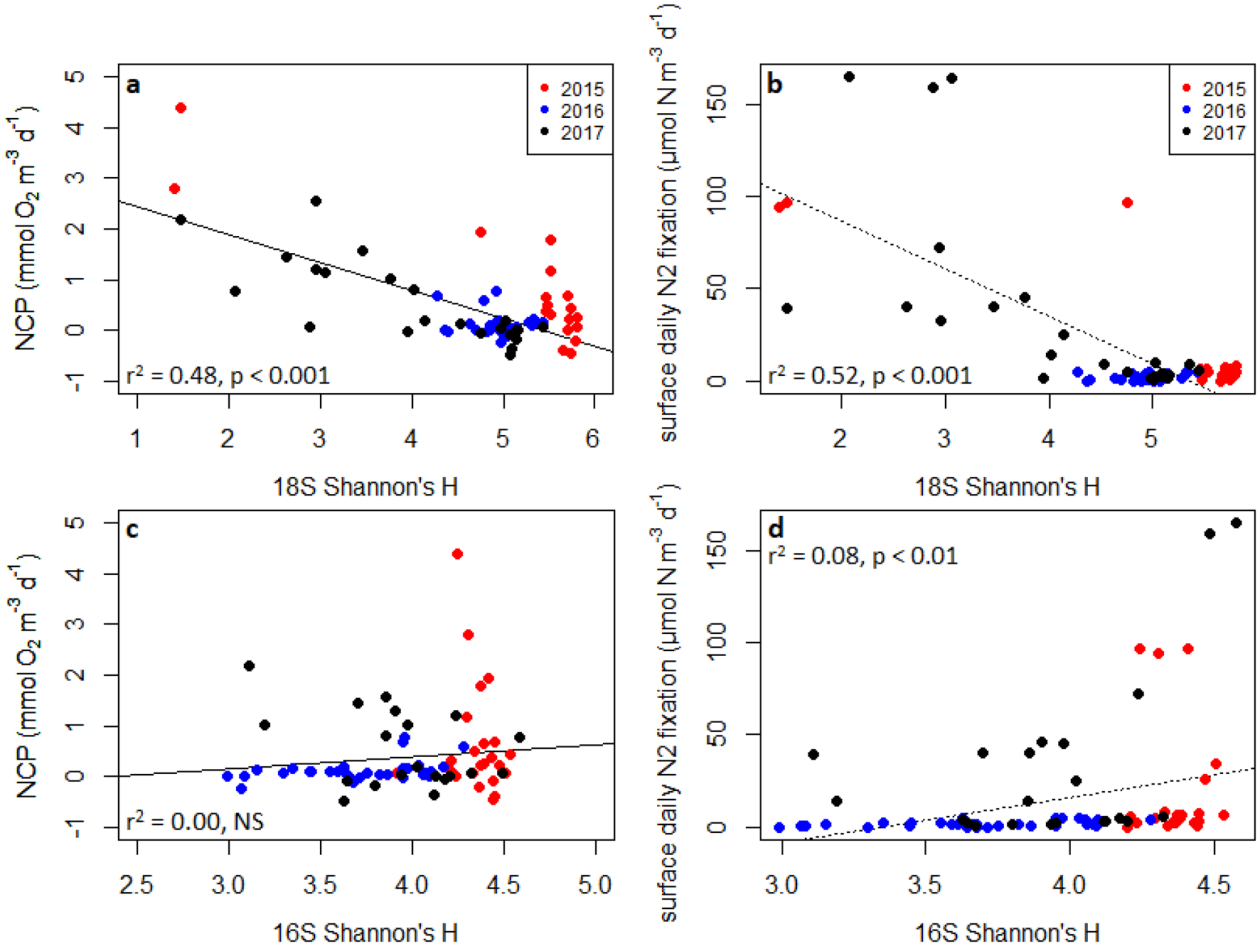
Linear regressions of (**a)** NCP and (**b)** N_2_ fixation rates vs 18S Shannon’s H diversity. Linear regressions of (**c)** NCP and (**d)** N_2_ fixation rates vs 16S Shannon’s H diversity. Significance and goodness-of-fit are displayed for each plot. All diversity metrics were calculated at the OTU level. Figure created in R 3.5.2 (https://www.r-project.org/).

We observed a negative relationship between N_2_ fixation and eukaryotic community Shannon’s H diversity (Pearson = − 0.73, p < 0.001; Spearman’s rho = not significant), in which stations with lower eukaryotic diversity tended to exhibit higher N_2_ fixation rates (Fig. 5). A similar negative relationship was observed between NCP rates and eukaryotic diversity (Pearson = − 0.70, p < 0.001; Spearman’s rho = − 0.31, p < 0.05). These correlations appear to be driven by both by decreasing species richness and evenness, as samples drawn from locations with high N_2_ fixation and NCP rates tended to contain substantially fewer OTUs, often two standard deviations lower than the rest of the dataset (mean: 397, s.d.: 131), yet were also dominated by particular taxa such as *Chrysophyceae* and *A. anophagefferens*. We note that the linear regressions shown in Fig. 5 are included as visual aids, and not meant to assert that these relationships are necessarily linear.

Prokaryotic Shannon’s H diversity was weakly correlated to N_2_ fixation rates (Pearson = 0.3, p < 0.01; Spearman’s rho = 0.48, p < 0.01) but not with NCP (p > 0.05) (Fig. 5, Table S2). We also note that samples collected in 2016 typically exhibited a narrow range of eukaryotic diversity compared to the range observed for other years (Fig. 5) as well as generally similar eukaryotic community structure (Figs. 2, 3), whereas the range of prokaryotic diversity was relatively similar across all three years. Diversity patterns were also compared to other parameters including macronutrient concentrations, dissolved and particulate trace metals, and silica, with trends discussed in detail in the Supplementary Material.

These results are of interest for considering the ecological characteristics of the coastal hotspots of N_2_ fixation and NCP sampled during this survey. Productivity-diversity relationships in oceanic ecosystems can provide insight into ecological processes involved, such as competitive exclusion^44^, “kill-the-winner”^45^, and co-existence.

Increasing competitive exclusion driven by opportunistic bloom taxa has been proposed as an explanation for the negative slope portion of unimodal productivity-diversity relationships^46^. Earlier studies suggested a unimodal “humped” shape to such relationships, in which diversity increases with productivity, peaking at intermediate levels of production, then falling^47,48^. More recent work has not only documented a range of productivity-diversity relationships ranging from positive to negative to flat^49,50^, but has also raised methodological concerns, postulating instead that diversity may not be correlated with productivity, with rare taxa impacting species richness but not greatly influencing production^51^.

Our results, showing negative relationships with decreasing eukaryotic diversity for increasing N_2_ fixation and NCP, raise the possibility that competitive mechanisms could play a role in limiting eukaryotic diversity within the sampled coastal blooms. One potential mechanism is that the dominant eukaryotic plankton identified in our sampling, *Chrysophyceae*, and *Aureococcus*, can produce allelopathic compounds to inhibit the growth of algal competitors^34,52,53^. Given the record abundances of UCYN-A observed at these sites^37^, such patterns raise the possibility that UCYN-A and its host *Braarudosphaera bigelowii* can tolerate these chemicals. *Aureococcus* is adapted for low-light conditions and capable of leveraging diverse uptake pathways for dissolved organic carbon and nitrogen^54,55^ while *Chrysophyceae* can engulf particulate matter via phagotrophy^56,57^, potentially giving both groups of mixotrophic algae versatile capabilities for out-competing other taxa.

A positive relationship between N_2_ fixation and prokaryotic diversity could imply a greater availability of varied bacterial niches within N_2_ fixation hotspots, but the observed relationships in this study are too weak to confidently support such a hypothesis. An OTU-based definition for prokaryotic diversity is imperfect, given the ambiguity of the “species” concept for prokaryotes^58,59^. Observed relationships may depend on how diversity is defined and which functional groups (algae, free-living and particle-associated bacteria, viruses) are captured by field methods and considered part of the community. The observed relationships with eukaryotic diversity are also strongly driven by the samples taken in 2017 and 2015 off the New England Shelf, raising the possibility that patterns with diversity would shift with broader geographic sampling or data from summers in other years. Furthermore, our dataset remains too limited to investigate the possibility that underlying relationships are curvilinear as opposed to linear. Different taxonomic definitions, such as amplicon sequence variants^60^ could also affect biodiversity metrics, a topic that is beyond the scope of this study but worthy of further investigation.

### Fine-scale relationships: key community members associated with N_2_ fixation and NCP

**Figure 6:**
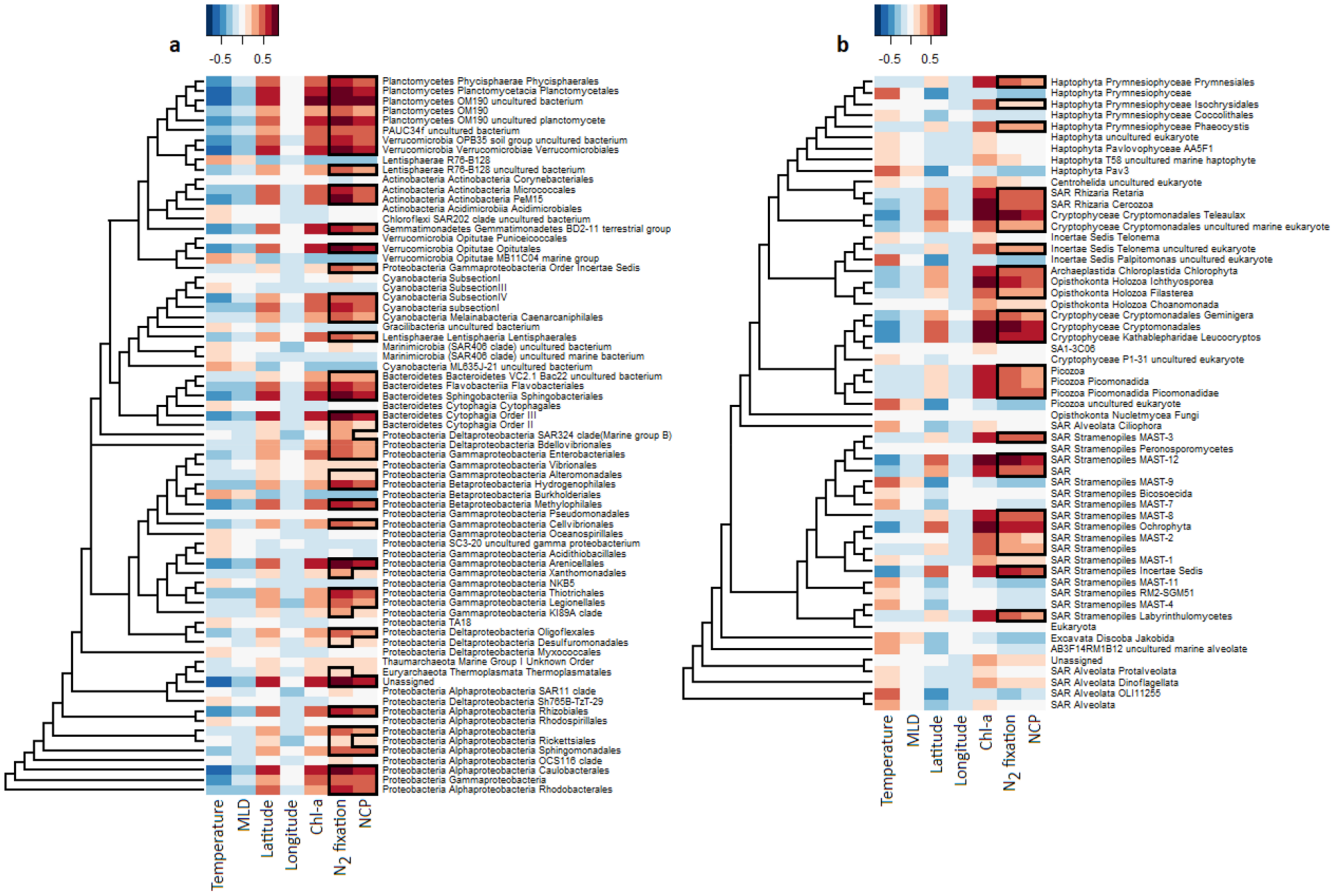
Heatmaps showing correlations between abundances of specific (**a)** prokaryotic 16S and (**b)** eukaryotic 18S taxa and measured metadata parameters for all three years of sampling, as determined using Partial Least Squares (PLS) regression analysis. The strength of correlations is illustrated by the color scale, with positive and negative correlations shown using warm and cool tones. Cells outlined in black rectangles indicate taxa with correlations to N_2_ fixation or NCP greater than 0.2. Taxa are binned at the fourth taxonomic rank for both 16S and 18S data. Only taxa for which the sum of corresponding reads is more than 0.5% of the total sum of all 18S or 16S reads are displayed. Dendrograms drawn to the left of each heatmap illustrate phylogenetic relationships between row taxa. Note that horizontal branch lengths have been compressed for layout reasons and no longer represent exact distances. Figure created in R 3.5.2 (https://www.r-project.org/).

Our partial least squares (PLS) regression analyses identified specific prokaryotic and eukaryotic taxa as jointly associated with high N_2_ fixation and high NCP rates (Fig. 6). Among prokaryotes, abundances for all *Planctomycetes* taxa binned at the fourth taxonomic rank were related to N_2_ fixation and NCP. 2 of 4 *Actinobacteria* taxa, 3 of 5 *Verrucomicrobia*, and 2 of 3 *Betaproteobacteria* also exhibited relationships with N_2_ fixation and NCP, as did *Arenicellales* (*Gammaproteobacteria*), *Sphingobacteriales*, and *Cytophagia* Order III from within *Bacteriodetes*. PLS regression analyses performed at the genus level identified a strong relationship between UCYN-A, a haptophyte-associated, N_2_ fixing group of unicellular cyanobacteria, and both N_2_ fixation and NCP (Dataset S1g).

*Planctomycetes* are largely heterotrophic, can exhibit surface and particle-associated lifestyles^61^ and have been observed at high abundances in coastal environments^62^. Consequently, relationships with productivity and diazotrophic activity could potentially reflect the particle-rich environment suggested by high quantitative abundances of *Chrysophyceae* and *Aureococcus anophagefferens*. A higher concentration of phytoplankton surfaces for growth might similarly explain N_2_ fixation and NCP associations displayed by *Bacteriodetes* and *Verrucomicrobia,* whose marine representatives are also typically particle-associated^63–65^. Alternatively, recent research indicates that heterotrophic bacterial diazotrophs, including some *Planctomycetes*, are more widespread and abundant in the surface ocean than previously thought^17^, potentially contributing directly to N_2_ fixation and thereby promoting new production.

Eukaryotic groups associated with both N_2_ fixation and NCP included most cryptophytes, *Ichthyosporea*, *Cercozoa*, *Prymnesiales*, *Retaria*, *Chlorophyta*, and several marine stramenopiles (MASTs) (Fig. 6). The taxon *Ochrophyta*, containing the dominant stramenopiles within the coastal transect—*Chrysophyceae* and *Aureococcus anophagefferens*—was also predictably related to NCP and N_2_ fixation. At the genus level, UCYN-A’s hypothesized prymnesiophyte host, *Braarudosphaera bigelowii*, was related to N_2_ fixation and NCP (Dataset S1h). PLS regression analyses of taxonomic abundances in relation to macronutrients, trace metals, and silica are shown in the Supplementary Material.

**Figure 7:**
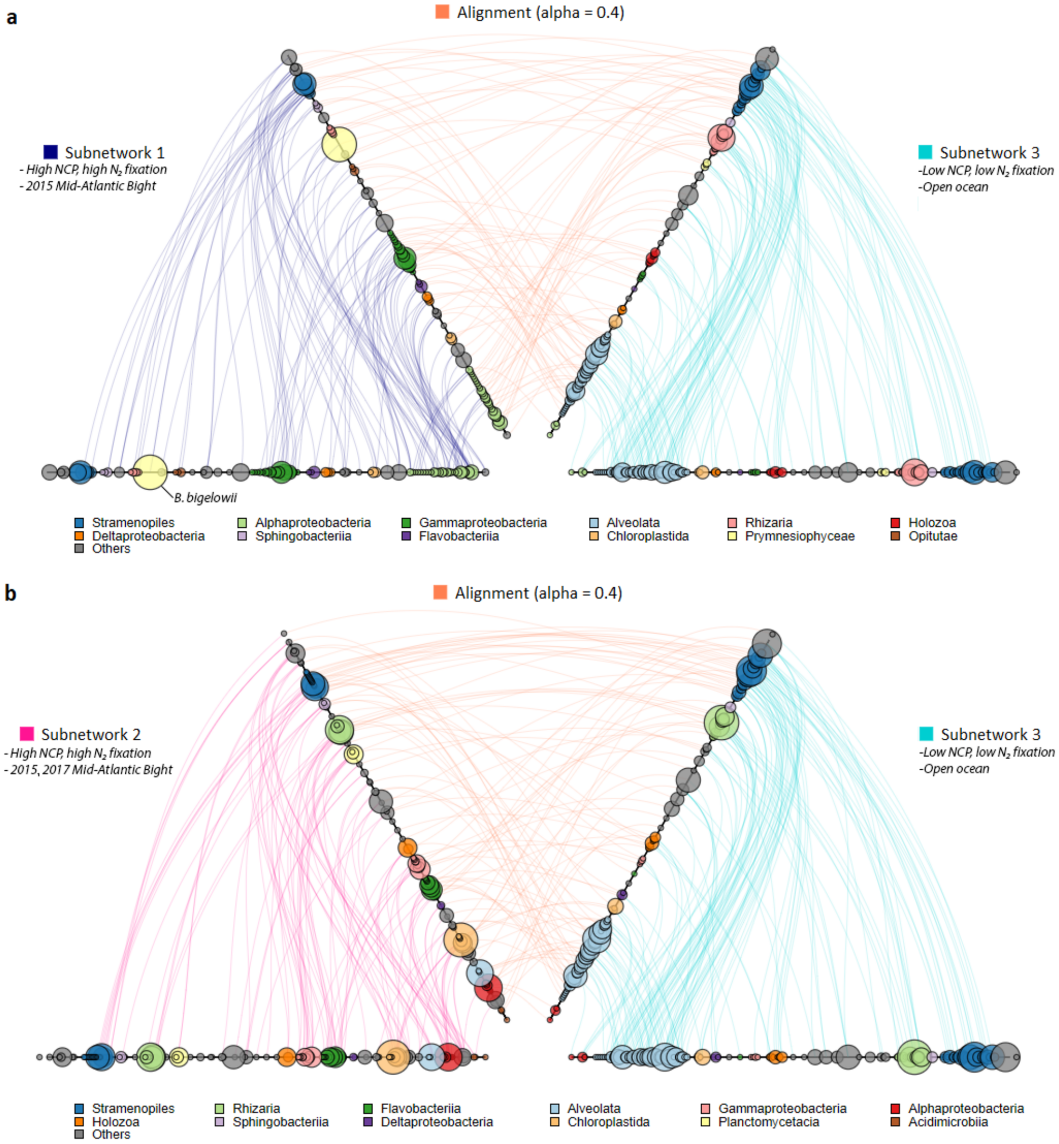
Alignment plots from co-occurrence and graph alignment analyses illustrate differences in ecological patterns and relationships between low N_2_ fixation + Low NCP and high N_2_ fixation + high NCP subnetworks. (**a)** Visualization of network relationships within and contrasted between Subnetwork 1 (2015 MAB, high N_2_ fixation) and Subnetwork 3 (open ocean, low N_2_ fixation) (**b)** and Subnetwork 2 (2015 + 2017 MAB, high N_2_ fixation) and Subnetwork 3 (open ocean, low N_2_ fixation). The leftmost pair of identical axes represent taxa belonging to the first subnetwork, with edges between the left two axes signifying significant co-occurrence relationships between taxa within the subnetwork. The rightmost axes and lines similarly represent taxa from the second subnetwork and within-network co-occurrence relationships. Edges between the two networks represent aligned taxa, where an OTU shares a similar taxonomic identity (sequence similarity) and a high degree of network topological similarity with an OTU in the other subnetwork. Alignments suggest that OTUs fill similar ecological roles within their respective subnetworks. Circular nodes represent individual OTUs, colored and grouped at the third taxonomic rank. Larger circle diameter indicates higher network importance (centrality). Taxa are ordered by centered log-ratio (CLR) normalized abundance, with higher-abundance genera placed further from the origin. Figure produced using HiveAlign (https://univ-nantes.io/erwan.delage/HiveAlign).

Network and graph-alignment analyses revealed several eukaryotic taxa associated with N_2_ fixation and NCP as hub organisms within their subnetworks or communities. These hub taxa showed high numbers of significant co-occurrence relationships, implying that they could play central community roles (Fig. 7). For example, Subnetwork 1 in Fig. 7 described a community associated with high N_2_ fixation and productivity sites in the 2015 coastal bloom (Fig. S3). Within this community, network analysis identified marine stramenopiles as important hub taxa, with four stramenopile genera including two chrysophytes placing among the top 10 keystone nodes. Notably, our results highlighted *Braarudosphaera bigelowii* as the taxon with the single highest network centrality within its N_2_ fixation and NCP-associated subnetwork, suggesting that this diazotroph-hosting prymnesiophyte may be ecologically influential. Taxa with high network centrality are critical to subnetwork stability by exhibiting a high number of connections with other OTUs. Other marine microbiome studies along the California coast^66^ and off Brazil^67^ have also found evidence that *B. bigelowii* may play important ecological roles within other microbial ecosystems. Equally interesting were taxa associated with N_2_ fixation and NCP in PLS regression analysis but exhibiting few sub-network connections, such as cryptophyte algae and *Aureococcus*. Their elevated abundance in productive samples but low participation in network structure could potentially suggest that cryptophytes and *Aureococcus* benefit opportunistically from ecosystem conditions but possess fewer direct interactions with other microbes in their environment.

Subnetwork 2 described a community generally associated with coastal sampling in 2015 and 2017 (Fig. S3). A similar centrality analysis revealed several chlorophyte genera—*Micromonas*, *Bathycoccus*, and *Ostreococcus*—as important keystone nodes. High network centrality associated with these taxa could conceivably reflect a potential role of these small, genetically-streamlined chlorophytes as prey within the community for larger mixotrophic and heterotrophic protists and a shared response alongside other taxa to favorable environmental conditions. That said, possibilities exist for more complex interactions—members of *Micromonas* in polar waters have recently been identified as mixotrophs, practicing bacterivory^68^. Our data cannot directly provide insight into these hypotheses. However, the high network centrality of chlorophyte taxa and their abundance in high N_2_ fixation and NCP samples suggest that they may be ecologically important members of the productive coastal community sampled.

Stramenopiles, including several *Chrysophyceae*, were also identified as essential taxa within the low N_2_ fixation, low-productivity Subnetwork 3 together with alveolates, driving much of the network structure within this module. To emphasize common patterns between stramenopiles in these subnetworks, we now examine the results of our graph alignment analysis. A graph alignment consists of identifying OTUs from two distinct communities that possess (i) similar marker gene sequences (i.e., sequence identity) and (ii) similar roles within the communities’ topological structure (i.e., patterns of centrality). Aligned OTUs from different subnetworks imply similar ecological roles in their respective communities. A large fraction of the significant alignments between Subnetwork 3 and Subnetworks 1 and 2 involved stramenopiles and alveolates from Subnetwork 3. This alignment result suggests that a community transition between these conditions could be associated with the resilience of these groups of eukaryotes and their related ecological functions.

Our overall findings corroborated this result. The observed close correspondence between peaks in N_2_ fixation and *Chrysophyceae* abundances supports the theory that stramenopiles potentially play a key role in our study region. However, this also highlights our lack of general understanding of the function and role of stramenopiles. To our knowledge, the oceanographic literature so far does not record any known direct association between *Chrysophyceae* and diazotrophs that could drive such a pattern, motivating the proposal of hypotheses to drive further studies. *Chrysophyceae* and diazotrophic activity could simply co-vary in response to a separate stimulatory factor. Alternatively, diazotrophic inputs of N might, through recycling via the microbial loop, act to promote *Chrysophyceae* growth. Mixotrophic *Chrysophyceae* possess diverse metabolic capabilities including heterotrophic uptake of organic matter using phagotrophy, potentially supporting such a hypothesis^56,57^. High abundances of *Chrysophyceae* might, on the other hand, create favorable conditions for UCYN-A and its host *B. bigelowii* through the competitive exclusion of competitors. Or, a direct interaction between *Chrysophyceae* and yet-uncharacterized N_2_-fixers could represent an unlikely but intriguing possibility. At any rate, this pattern of association between *Chrysophyceae* and high measured N_2_-fixing activity warrants a more detailed investigation driven by interaction studies.

## Conclusion

Our study of prokaryotic and eukaryotic community structure within hotspots of N_2_ fixation and NCP revealed an intriguing microbial ecology. We observed a strong negative relationship between eukaryotic diversity and both N_2_ fixation and O_2_/Ar-derived NCP, a finding suggesting that low eukaryotic microbial diversity at productive coastal sites might potentially be driven by competitive exclusion. Notably, we observed high abundances of *Chrysophyceae* occurring in strikingly close conjunction with high N_2_ fixation rates and NCP*,* along with elevated abundances of *Aureococcus anophagefferens*, chlorophytes, cryptophytes, and *Planctomycetes*. Such a community structure indicates that observed high N_2_ fixation rates may be occurring in microbial ecosystems with a high potential to process and take up diverse forms of organic material and nutrients. Aided by our quantitative study design, our network analysis highlighted *Braarudosphaera bigelowii*, chlorophytes, and *Chrysophyceae* and other marine stramenopiles as potentially important community hub taxa. Overall, our findings point to notable taxa driving the ecological network structure of coastal N_2_ fixation hotspots and, particularly in light of the establishment of a new LTER site along the northeast U.S. coastal shelf (NES-LTER)^69^, can serve as a springboard for targeted investigations into the processes mediated by these microorganisms of interest.

## Materials and methods

### Underway measurements of N_2_ fixation rates and net community production

Our combined set of new and previously-published data includes around 10,250 km of continuous and discrete measurements across the western North Atlantic Ocean over three summer voyages aboard the *R/V Atlantic Explorer*. Previously-reported surface layer N_2_ fixation rates^22,37^ were measured at high resolution using the Flow-through incubation Acetylene Reduction Assay by Cavity ring-down Spectroscopy (FARACAS)^38^, which utilizes a cavity ring-down spectrometer to measure the conversion of dissolved acetylene to ethylene by diazotrophs within surface seawater. Previously-reported measurements of NCP^22,36,37^ were conducted using the dissolved O_2_/Ar method via Equilibrator Inlet Mass Spectrometry^70^. Detailed protocols and data analysis procedures can be found in previous publications^22,36,37^. We conducted a sensitivity analysis to assess the effect of vertical O_2_/Ar fluxes upon calculated NCP rates, detailed in the Supplementary Methods, which we deemed to be minimal. The cruise tracks, conducted from 3 to 13 August 2015, 3–12 August 2016, and 29 July-7 August 2017, are illustrated in Fig. 1, with associated physical and biogeochemical properties shown in Fig. S1 and S2. Our N_2_ fixation technique calculates daily fixation rates, while NCP measurements integrate over 2–8 days, both reasonable timescales for comparisons between community structure and these rate measurements.

### Sample collection and library preparation for 16S and 18S rDNA amplicon sequencing

Microbial community composition data from 2015 was previously published in Wang et al., 2018^36^, whereas molecular data and samples from the 2016 and 2017 are original to this paper. Molecular samples for rDNA amplicon sequencing were collected every 6–8 h using 0.22 μm polycarbonate filters (Millipore, Billerica, MA, USA) from surface (3–5 m) seawater collected from towfish^71^ and 5 m CTD samples. Between 1–2 l (mean: 1.2 l) were filtered using a peristaltic pump, with smaller volumes filtered at particularly high-biomass stations. In 2015, due to the leveraging of samples initially collected for RNA sequencing, all utilized filters were preserved in RNAlater (Thermo Fisher, Waltham, MA, USA), while RNAlater preservation was not employed for 2016 and 2017 samples. Otherwise, the sampling methodology was identical across all three years. Filters were flash-frozen in liquid nitrogen following filtration and stored at − 80 °C.

The addition of internal standard control sequences was performed following the protocol described in Wang et al., 2018^36^ for all samples. For the 2016 and 2017 samples, new standard stock solutions were prepared and amounts added were adjusted based on the 2015 sequencing results. *T. thermophilus* gDNA standard was added to each sample before bead-beating in 50 μl volumes with amounts of 2,800,000 copies (3.05 ng) per sample for 2015 samples and 14,040,000 copies (15.25 ng) per sample for 2016 samples. For both 2015 and 2016 samples, 5,780,000 copies (0.68 ng) of *S. pombe* gDNA were introduced. For 2017 samples, 0.66 ng of *S. pombe* gDNA and 6.1 ng of *T. thermophilus* gDNA were added, corresponding to approximately 5,610,000 copies sample^−1^ and 5,620,000 copies sample^−1^ respectively.

DNA extractions, PCR amplification of the 16S rDNA V4 and the 18S rDNA V4 regions, sample quantitation, and library pooling followed the same procedure as Wang et al., 2018^36^. Briefly, DNA extraction was performed using the Qiagen DNeasy Plant Mini Kit, utilizing the manufacturer’s instructions with slight modifications^72^. PCR parameters were as follows for 16S samples: an initial denaturing step of 94 °C for 3 min, followed by 30 cycles of 94 °C for 30 s, 60 °C for 30 s, 72 °C for 1 min, and ending with a final elongation step at 72 °C for 10 min. 18S parameters were identical apart from an annealing temperature of 57 °C. The PCR step employed the custom 16S rDNA V4 primers 515F-Y (5′-GTGYCAGCMGCCGCGGTAA-3′) and 805 R (5′-GACTACNVGGGTATCTAAT-3′) and 18S rDNA V4 primers F (5′-CCAGCASCYGCGGTAATTCC-3′) and R (5′-ACTTTCGTTCTTGAT-3′), with attached Illumina adapters and barcodes^36^. 16S PCR reactions (25 µl volume) contained 2.5 µl 10 × PCR buffer, 0.5 µl dNTP mix (10 mM each), 1 µl 50 mM MgSO_4_, 0.5 µl of each primer (10 µM), 0.1 µl Platinum Taq Hi-Fidelity Polymerase, and 19.4 µl of sterile water, plus 0.5 µl of template. 18S PCR reaction mixtures were identical, although template and polymerase amounts were adjusted (0.1–0.2 µl polymerase, 0.5–3 µl template) with corresponding adjustments to sterile water volume to address weak amplification of some samples. PCR products were purified with the Qiagen QIAquick PCR Purification Kit, quantified using a Qubit 3.0 fluorometer, then pooled in equimolar amounts into a 16S and an 18S library. Illumina MiSeq (300 bp PE, V3) sequencing was subsequently performed at the Duke Center for Genomic and Computational Biology.

### rDNA sequencing data processing and OTU picking

We obtained 43,258,158 paired-end reads from 167 surface samples. Paired-end sequences were trimmed, merged, quality filtered, demultiplexed, then filtered for chimeras as in Wang et al., 2018^36^, yielding 11,474,817 16S sequences and 4,988,755 18S sequences. Primer and other non-biological sequences were then filtered using Tagcleaner^73^. Open-reference OTU picking was subsequently performed in QIIME (97% similarity, Usearch 6.1^74^, SILVA 123.1^75^)^36^ (see Supplementary Material for additional details). Following taxonomy assignment, we removed low read count samples (< 100 sequences) and filtered internal standard, plastid, mitochondrial, and metazoan rDNA sequences from our taxonomy tables. Phylogenetic trees were generated using the make_phylogeny.py script in QIIME, which utilizes the FastTree method.

### Statistical analyses

The combined 2015–2017 sequencing dataset included 8841 16S OTUs across 79 16S samples and 14,344 18S OTUs across 79 18S samples, post-filtering. Sequencing data were imported into R 3.4.1^76^ using the phyloseq^77^ package. Calculation of quantitative abundances was performed as in Satinsky et al., 2013^78^, with the total rDNA gene abundance l^-1^ of a taxon determined as the number of reads mapping to that OTU in the sequencing output, divided by the product of the standard recovery ratio (# standard reads recovered / # standard reads introduced) and the volume filtered. The internal standard recovery ratios obtained in this study are shown in (Fig. S4). Details of filtration of low-count samples and outliers and rarefaction and ordination procedures are described in the Supplementary Material.

The internal standard technique has previously demonstrated good agreement with other quantification techniques including flow cytometry counts, HPLC, phospholipid fatty acid (PLFA) analysis, and substrate-induced respiration (SIR) analysis^79–81^. However, the approach remains subject to limitations. This quantitative method does not account for variable rDNA gene copy number across bacterial and eukaryotic taxa. Prokaryotic rDNA copy numbers mostly vary between 1 and 15 copies per cell^82^, while eukaryotic rDNA gene copies cell^-1^ can vary by orders of magnitude. Additionally, the internal standard technique assumes that recovery rates are identical for internal standard reads and natural sequences, an assumption that primer, amplification, or DNA extraction biases could impact. Consequently, calculated abundances should not be interpreted as absolute estimates of organismal cell concentrations, but rather reflect the changing quantitative abundance of taxa across samples.

Calculation of quantitative 16S rDNA gene abundances produced a discrepancy in which samples collected in 2015 displayed significantly higher total 16S rDNA abundances (Wilcoxon rank-sum test, p < 0.001) than 16S rDNA samples obtained in 2016 and 2017. This difference in calculated abundances likely results from quantitation or dilution errors introduced while synthesizing the varying 16S spike quantities and stock solutions between years as mentioned above and from differences in preservation protocols. To investigate this, we compared our calculated SAR11 rDNA copy numbers for stations within 400 km of Bermuda against reported summer SAR11 abundances of 1.5–2.0 × 10^8^ cells/l for this region^83,84^. While calculated 2016 and 2017 SAR11 abundances (1.41 × 10^8^ cells/l and 1.38 × 10^8^ cells/l) aligned closely with values from the literature, samples from 2015 near Bermuda displayed a median SAR11 abundance of 6.5 × 10^8^ cells/l. Consequently, we adjusted calculated 2015 16S rDNA abundances by a factor of 0.21. Adjusted 2015 organismal abundances for SAR11 and *Prochlorococcus* align closely with expected abundances from the literature (Table S2). We further cross-validated our corrected organismal abundances for *Prochlorococcus* against patterns of *Prochlorococcus* abundance obtained from concurrently-collected flow cytometry samples, observing good agreement in abundance patterns between the two datasets (Table S1, Fig. S5). The corrected 2015 16S rDNA dataset was subsequently used alongside the 2016 and 2017 datasets in generating taxonomy plots, ordination analyses, Partial least squares (PLS) regression analyses, and co-occurrence network construction.

Partial least squares (PLS) regression analysis was performed following Wang et al., 2018^36^ upon 18S and 16S taxa at the fourth and sixth taxonomic ranks. The fourth taxonomic rank was the highest resolution for which heatmap figures are readily visually interpretable, while the sixth (genus) rank is the highest taxonomic level that the 18S and 16S rDNA amplicon can accurately and reliably resolve. Taxa at the fourth and sixth taxonomic ranks with read counts comprising < 0.5% of the total sum of 18S or 16S reads were filtered from analyses. Two separate PLS regression analyses were performed: one utilizing the full molecular dataset from all 3 years to assess taxon-specific relationships with NCP and N_2_ fixation, and another utilizing only the community data from the subset of samples collected in 2016 and 2017 to investigate relationships between specific taxa and macronutrient and trace metal concentrations (see Supplementary Material). To handle missing data, the non-iterative partial least squares (nipals) algorithm was employed.

### Network analysis

Weighted gene correlation network analysis (WGCNA)^85^ was performed to select subnetworks of co-occurring marine microbial OTUs from the whole dataset. Following the WGCNA standard protocol^86^, we constructed an adjacency matrix of the microbial community dataset using Pearson correlations (R^2^ cutoff = 0.85)^87^. A soft-thresholding power law (p = 8) was applied to fit the weighted graph into a scale-free topology. A topological overlap measure (TOM) was then calculated for each pair of OTUs, based on the weight of their pairwise correlations with close neighbors and weighted correlations with other OTUs. Finally, hierarchical clustering based on TOM was performed to identify sub-networks of co-occurring taxa. Two sub-networks (Subnetworks 1 and 2) that were most significantly positively associated with both NCP and N_2_ fixation (Figs. S3, S6) were extracted, as well as the subnetwork (Subnetwork 3) most negatively correlated with these rates, although not significantly.

A global, robust network was built via FlashWeave^88^. Induced subgraphs, as identified from WGCNA, were then extracted. Subnetwork comparison was then performed using L_GRAAL, a graph alignment tool^89^. L_GRAAL aligns OTUs from two distinct co-occurrence networks when they share (i) similar topological properties and (ii) rDNA sequence homology (alpha = 0.4 for accurate consensus between both features)^90^. Visualization of subnetworks and alignments was performed with HiveAlign. More details on specific steps taken for WGCNA, Flashweave, and L-GRAAL analyses can be found in the Supplementary Material.

## Supplementary Information

Supplementary Information.
