# Supplementary material for: Investigating the microbial ecology of coastal hotspots of marine nitrogen fixation in the western North Atlantic
Source: Sci Rep. 2021 Mar 9;11:5508. doi: 10.1038/s41598-021-84969-1 (PMC7943828; doi:10.1038/s41598-021-84969-1)
Supplement: Supplementary file 1 — Supplementary Information. [file 41598_2021_84969_MOESM1_ESM.docx]

**Supplementary Information for:**

**Investigating the microbial ecology of coastal hotspots of marine nitrogen fixation in the western North Atlantic**

Seaver Wang^a^, Weiyi Tang^b^, Erwan Delage^c^, Scott Gifford^d^, Hannah Whitby^e^, Aridane G. González^f,g^, Damien Eveillard^c^, Hélène Planquette^g^, Nicolas Cassar^a,g^*

^a^Division of Earth and Ocean Sciences, Duke University, Durham, NC, USA.

^b^Department of Geosciences, Princeton University, Princeton, NJ, USA.

^c^Université de Nantes, CNRS, UMR 6004, LS2N, F-44000 Nantes, France.

^d^Department of Marine Sciences, the University of North Carolina at Chapel Hill, Chapel Hill, NC, USA.

^e^Department of Earth, Ocean, and Ecological Sciences, School of Environmental Sciences, University of Liverpool, Liverpool, UK.

^f^Instituto de Oceanografía y Cambio Global (IOCAG), Universidad de Las Palmas de Gran Canaria, ULPGC.

^g^Laboratoire des Sciences de l'Environnement Marin (LEMAR), Institut Universitaire Européen de la Mer (IUEM), Technopoôle Brest-Iroise, 13 Plouzaneì 29280, France

***Corresponding author:**

Professor Nicolas Cassar
Box 90328, Grainger Environment Hall, Duke University

9 Circuit Drive
Durham, NC 27708
[nicolas.cassar@duke.edu](mailto:seaver.wang@duke.edu)

(919) 681-8865

**Supplementary Figures**


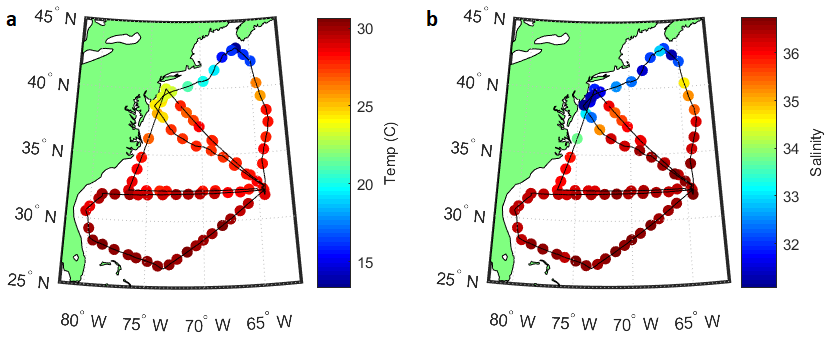


**Fig. S1** **a** Temperature and **b** Salinity measurements recorded at sampling stations across July-August 2015, 2016, and 2017 cruises in the Western North Atlantic^1,2^. Plots created using Matlab 2018b (<https://www.mathworks.com/products/matlab.html>).


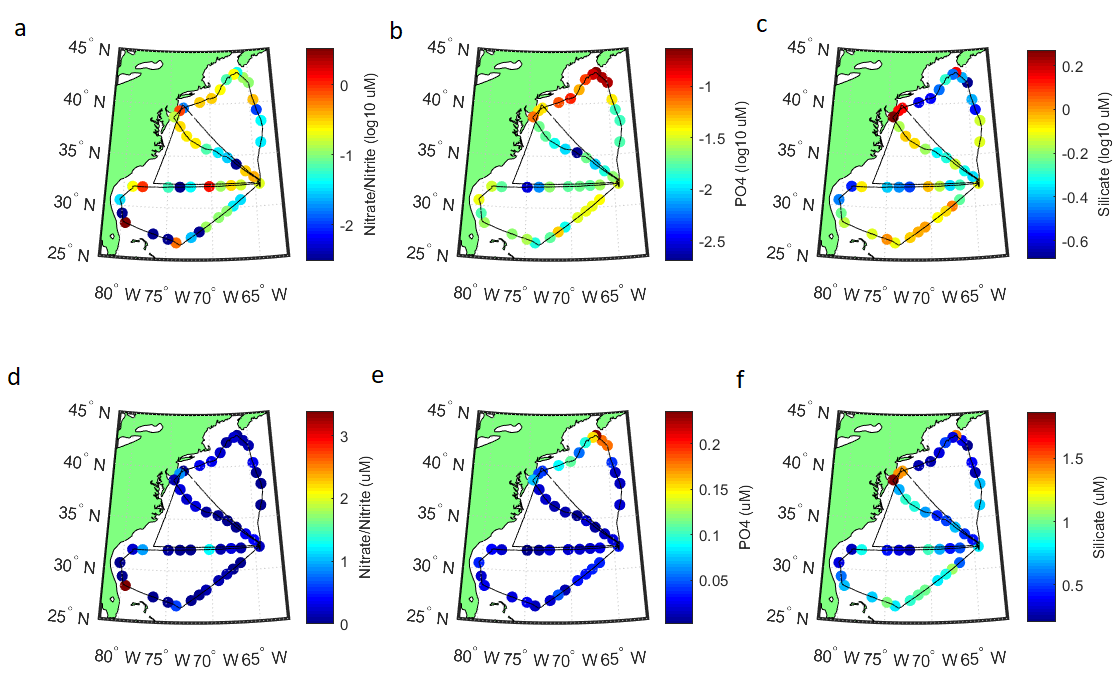


**Fig. S2** **a** log NO_2_+NO_3,_ **b** log PO_4_, and **c** log silicate concentrations across the 2016 and 2017 cruises^1,2^. **d** NO_2_+NO_3_, **e** PO_4_, and **f** silicate concentrations. Plots created using Matlab 2018b (<https://www.mathworks.com/products/matlab.html>).


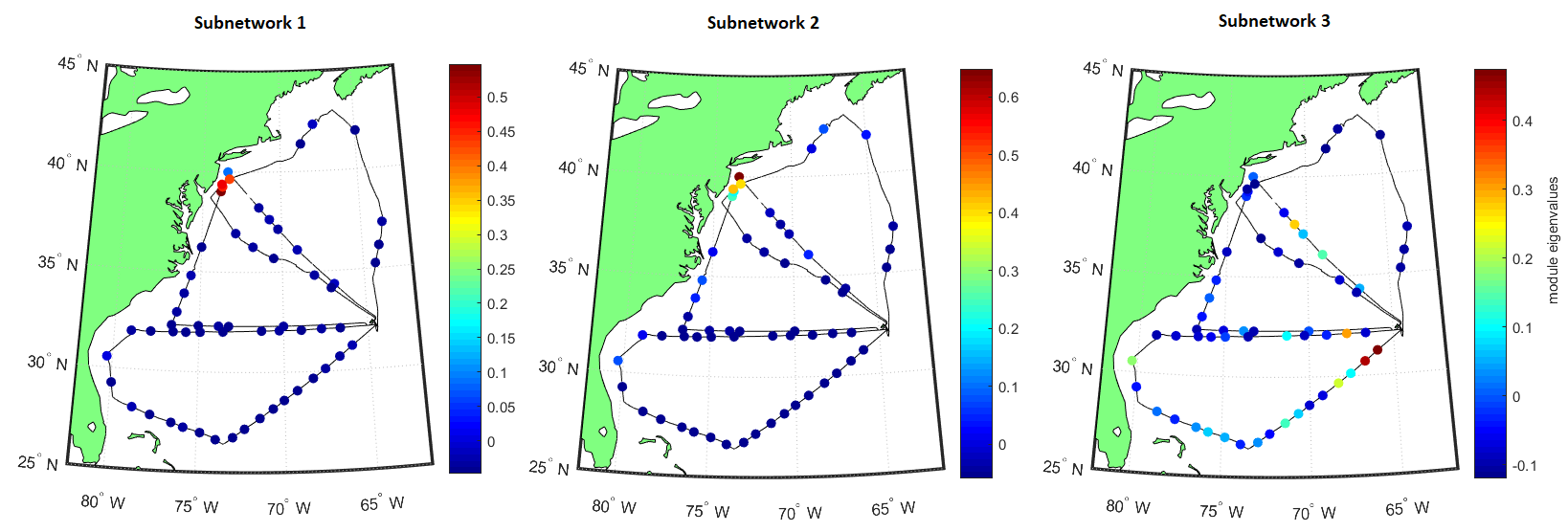


**Fig. S3** Maps showing the strength of associations between each WGCNA-derived module and the sampled sites. Subnetwork 1 (2015 MAB, high N_2_ fixation), Subnetwork 2 (2015+2017 MAB, high N_2_ fixation) and Subnetwork 3 (open ocean, low N_2_ fixation). The color of each point represents the magnitude of the eigenvalue for that module corresponding to the sampled station. Plots created using Matlab 2018b (https://www.mathworks.com/products/matlab.html).


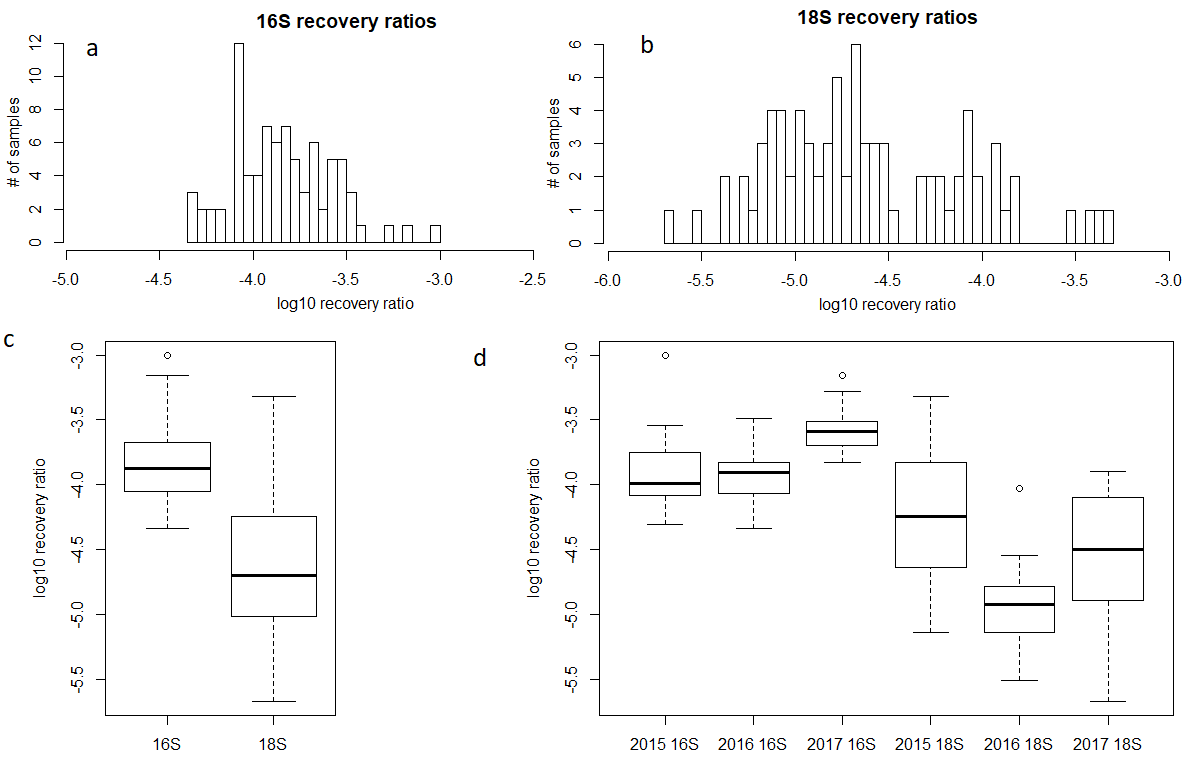


**Fig. S4** Histograms of the distribution of log-scaled internal standard recovery ratios for **a** 16S rDNA and **b** 18S rDNA molecular samples. Box-and-whisker plots of log-scaled recovery ratios for **c** the overall 16S rDNA and 18S rDNA datasets and **d** 16S rDNA and 18S rDNA samples subdivided by year. Box edges are defined by the first and third quartiles of the data. Individual points are shown only if identified as outliers, located >1.5x the full length of the box away from the nearest edge of the box. Whiskers are positioned at the lowest and highest values that are not identified as outliers. Figure created in R 3.5.2 (https://www.r-project.org/).


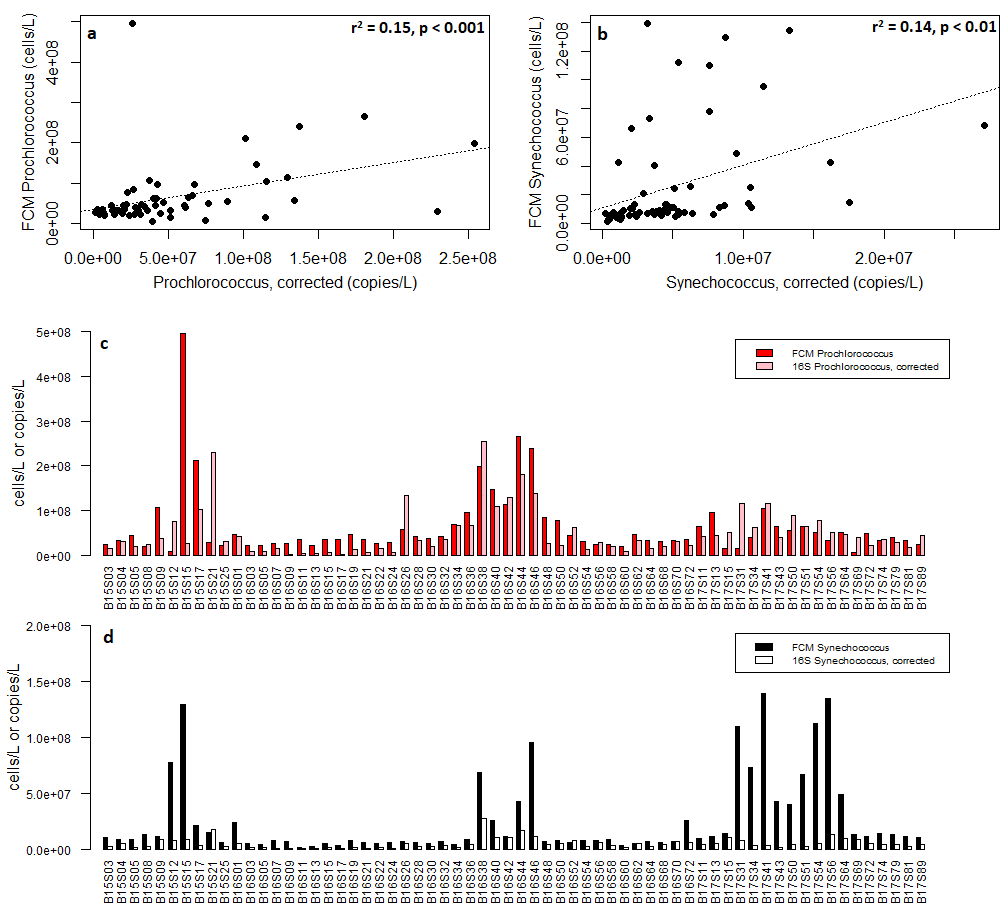


**Fig. S5** Scatter plot and goodness-of-fit of relationships between **a** *Prochlorococcus* and **b** *Synechococcus* flow cytometry cell counts and corrected quantitative 16S rDNA amplicon abundances. Barplots illustrating abundances of **c** *Prochlorococcus* and **d** *Synechococcus* as determined using flow cytometry cell counts and (corrected 2015) 16S rDNA gene abundances across the three-year sample dataset. Figure created in R 3.5.2 (https://www.r-project.org/).


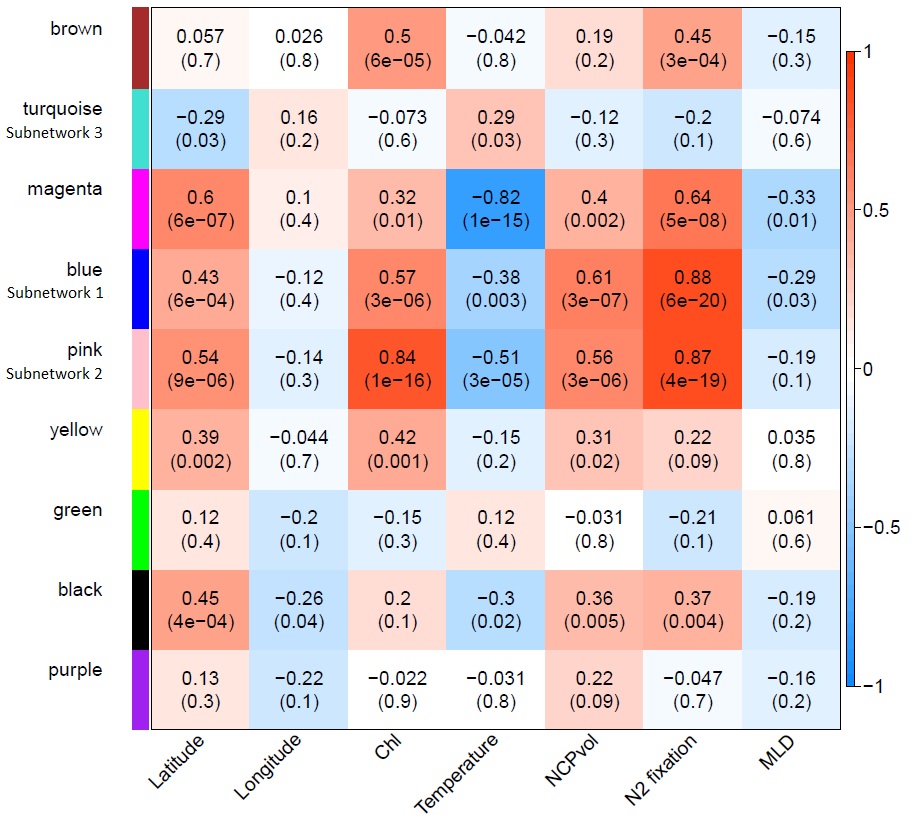


**Fig. S6** Heatmap illustrating correlations between WGCNA modules and metadata parameters. Positive correlations are denoted using a warm color scale, while negative correlations are shown in cool colors. Pearson correlation coefficients are given in each cell, with p-values shown in parentheses below. Figure created in R 3.5.2 (https://www.r-project.org/).

**
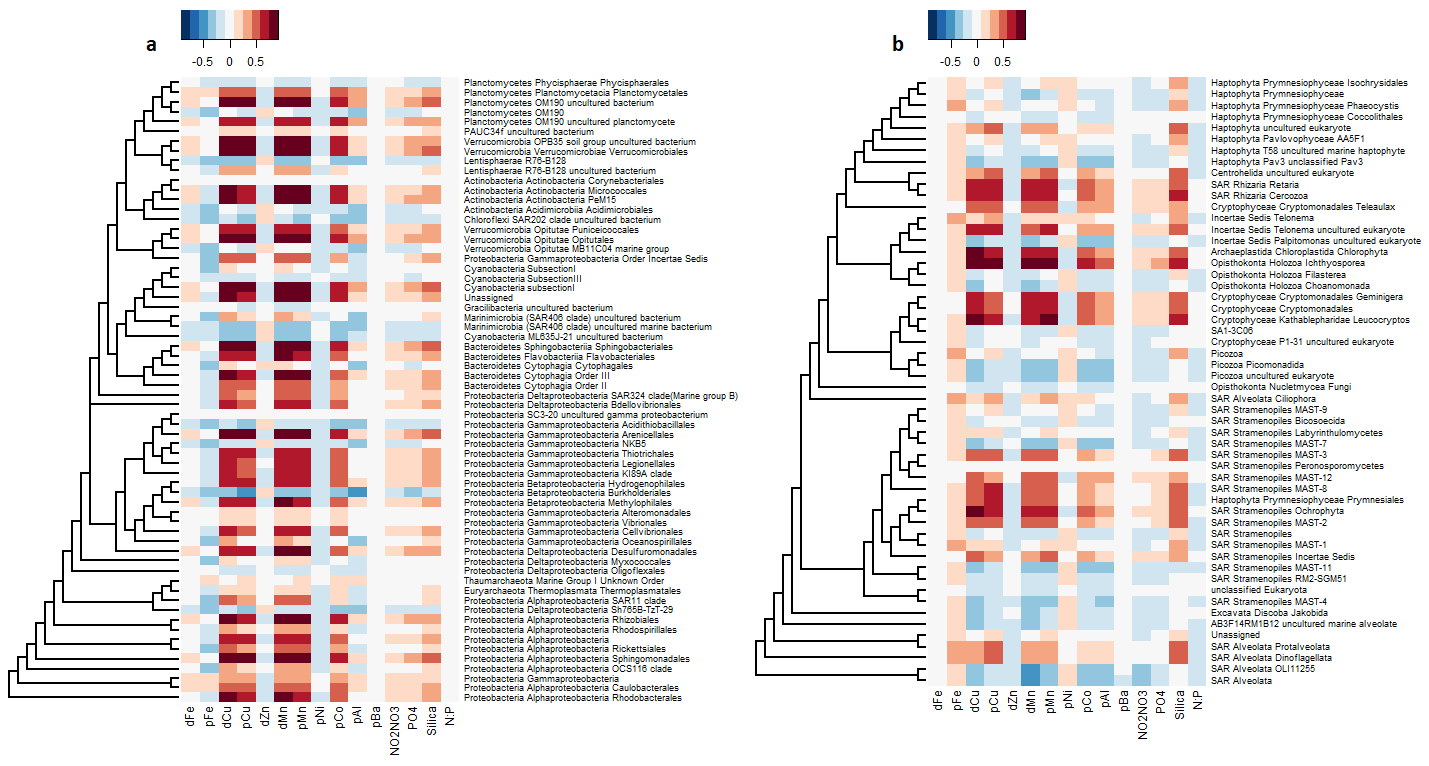
**

**Fig.** **S7** Heatmaps showing PLS-derived correlations between abundances of specific **a** prokaryotic 16S and **b** eukaryotic 18S taxa and measured trace metal and nutrient concentrations from 2016 and 2017. Strength of correlations is illustrated by the color scale, with positive and negative correlations shown using warm and cool tones. Taxa are binned at the fourth taxonomic rank for both 16S and 18S data. Only taxa for which the sum of corresponding reads are more than 0.5% of the total sum of all 18S or 16S reads are displayed. Dendrograms drawn to the left of each heatmap illustrate phylogenetic relationships between row taxa. Note that horizontal branch lengths have been compressed for layout reasons and no longer represent meaningful distances. Figure created in R 3.5.2 (https://www.r-project.org/).

**Table S1** Comparison of *Prochlorococcus*, *Syncechococcus*, and SAR11 cell concentrations from the literature and from flow cytometry samples with quantitative *Prochlorococcus*, *Syncechococcus*, and SAR11 16S rDNA gene abundances.

| **16S abundances, this study** | **Pro median copies/L** | **Syn median copies/L** | **SAR11 median copies/L** | **Notes** |
| --- | --- | --- | --- | --- |
| **2015** | 1.70E+08 | 1.85E+07 | 6.50E+08 | Samples <400km from Bermuda |
| **2016** | 1.70E+07 | 3.29E+06 | 1.41E+08 | Samples <400km from Bermuda |
| **2017** | 2.15E+07 | 2.90E+06 | 1.38E+08 | Samples <400km from Bermuda |
| **corrected 2015** | 3.58E+07 | 3.89E+06 | 1.36E+08 |  |
|  |  |  |  |  |
|  |  |  |  |  |
|  | **Pro (cells/L)** | **Syn (cells/L)** | **SAR11 (cells/L)** | **Notes** |
| **Morris et al 2002** |  |  | 1.6E+08 to 2E+08 | August 2001 vicinity of Bermuda |
| **Carlson et al 2008** |  |  | 1.50E+08 | Summer, BATS |
| **Zinser et al 2006 AEM** | 5E+07 to 1E+08 |  |  | August 2002 near Bermuda |
| **Zinser et al 2007** | 3E+07 to 6E+07 |  |  | late August near Bermuda |
| **Singh et al 2015** | 1E+07 to 2E+07 | 1.00E+07 |  | Summer, BATS, multiple years |
| **Parsons et al 2012** | 2.5E+07 to 5E+07 | 1E+07 to 2E+07 | 1E+08 to 2E+08 | Summer, BATS, multiple years |
| **Range of FCM counts (this study)** | 2.2E+07 to 4.2E+07 | 8.1E+06 to 1.1E+07 |  |  |
|  |  |  |  |  |
| **Correction factor, SAR11** | **multiply 2015 by 0.21** |  |  |  |

**Table S2** Table of correlation coefficients for relationships between 16S rDNA and 18S rDNA Shannon’s H diversity and metadata variables. dFe, dCu, etc… denote dissolved trace metals. pFe, pCu, etc… represent particulate trace metals. Correlations are calculated for all three years of data for NCP, N_2_ fixation, Lat, Lon, Temperature, Salinity, Chl-a, and MLD. Correlations for macronutrients and micronutrients, which were only sampled in 2016 and 2017, are only made against molecular data from those years. Bold values significant at p<0.05.

|  | **Correlation w 18S Shannon diversity** | | | **Correlation w 16S Shannon diversity** | | |
| --- | --- | --- | --- | --- | --- | --- |
|  | Pearson | Spearman | Linear model R^2^ | Pearson | Spearman | Linear model R^2^ |
| **NCP** | **-0.7** | **-0.31** | **0.48** | 0.12 | 0.19 | 0.001 |
| **N_2_ fixation** | **-0.73** | -0.21 | **0.52** | **0.3** | **0.48** | **0.08** |
| **Latitude** | **-0.48** | -0.14 | **0.22** | **0.37** | **0.49** | **0.12** |
| **Longitude** | -0.004 | -0.03 | -0.01 | **-0.24** | **-0.26** | **0.05** |
| **Temperature** | **0.64** | **0.198** | **0.4** | -0.06 | **-0.45** | -0.01 |
| **Salinity** | **0.8** | 0.08 | **0.64** | -0.18 | **-0.64** | 0.02 |
| **N:P** | 0.14 | -0.06 | -0.004 | 0.17 | 0.18 | 0.003 |
| **NO_2_/NO_3_** | -0.024 | **-0.32** | -0.02 | 0.072 | 0.08 | -0.02 |
| **PO_4_** | **-0.56** | **-0.68** | **0.29** | -0.1 | -0.09 | -0.01 |
| **Silica** | -0.19 | 0.02 | 0.01 | 0.3 | 0.13 | 0.07 |
| **Chl-a** | **-0.46** | -0.05 | 0.2 | **0.26** | **0.62** | **0.06** |
| **MLD** | **0.47** | **0.37** | **0.21** | **0.22** | 0.13 | **0.04** |
| **dFe** | 0.18 | 0.24 | 0.19 | 0.09 | 0.045 | 0.54 |
| **pFe** | 0.07 | -0.04 | -0.02 | -0.18 | **-0.32** | 0.01 |
| **dCu** | **-0.81** | **-0.42** | **-0.64** | **0.29** | 0.12 | 0.06 |
| **pCu** | **-0.46** | **-0.61** | **0.2** | 0.1 | -0.23 | -0.02 |
| **dZn** | 0.16 | 0.25 | 0.01 | 0.11 | 0.22 | -0.01 |
| **dMn** | **-0.61** | **-0.34** | **0.36** | 0.28 | **0.36** | **0.06** |
| **pMn** | **-0.49** | **-0.49** | **0.23** | 0.3 | 0.16 | 0.07 |
| **pAl** | **-0.44** | -0.2 | **0.17** | 0.12 | -0.04 | -0.01 |
| **pBa** | -0.05 | -0.21 | -0.02 | 0.05 | 0.001 | -0.02 |
| **dCo** | -0.22 | -0.08 | 0.01 | 0.16 | 0.07 | -0.01 |
| **pCo** | **-0.62** | **-0.65** | **0.38** | 0.04 | -0.16 | -0.02 |
| **dNi** | **-0.85** | **-0.71** | **0.71** | -0.24 | -0.21 | 0.002 |
| **pNi** | 0.21 | -0.04 | 0.02 | -0.19 | -0.24 | 0.01 |

**Supplementary Material**

**Relationships between microbial abundances, diversity, and nutrient concentrations**

In addition to N_2_ fixation and NCP, this analysis also examined relationships between marine microbial community structure and measured concentrations of macronutrients as well as particulate (i.e. pFe) and dissolved (i.e. dFe) trace metals. Overall, low eukaryotic diversity is significantly associated with and high PO_4_, dCu, pCu, dMn, pMn, and pAl concentrations (p<0.05 for all) (Table S2). These trends are all significant when tested using alternative diversity metrics (Simpson, raw OTU richness: p<0.05 for all), demonstrating that the choice of diversity metric does not affect this pattern. Neither eukaryotic nor prokaryotic diversity show relationships with measured iron concentrations. Prokaryotes associated with N_2_ fixation and NCP also show strong associations with dCu, pCu, dMn, pMn, pCo, pAl, PO_4_, and Silica (Fig. S7).

The significant negative relationship between eukaryotic diversity and both dissolved and particulate Cu (Table S2) may suggest another potential exclusion-based mechanism. Elevated concentrations of free Cu are toxic to algae and cyanobacteria^3^, inducing production of protective organic ligands to bind free Cu and impacting cyanobacterial N_2_ fixation^4^. Cu concentrations observed at low-diversity stations were relatively high, with dCu reaching 4.1 nM^1^, similar to values considered toxic to the most sensitive marine microbial species^5^. Additionally, numerous species of algae release allelopathic toxins in response to Cu-induced toxicity stress^6-8^, possibly contributing to a reduction in biodiversity through competitive exclusion. We acknowledge that Cu toxicity depends on the bioavailability of Cu, linked to its chemical speciation and complexation with natural organic binding ligands, rather than the total metal concentration^9^. That said, Cu has been shown previously to impact the growth of cyanobacteria in this region^10^. The highest Cu concentrations are measured along the coast in our study, consistent with a terrestrial source. High pAl concentrations associated with these coastal sites also suggest strong lithogenic influences in the coastal region^11^. Consequently, a strong concentration gradient in dCu oriented along proximity to the coast could also mean that correlations with eukaryotic diversity, also distinct between the coast and open ocean, are tied to one or more other variables.

Ultimately, such corresponding patterns complicate attempts to attribute eukaryotic diversity patterns, high N_2_ fixation, or elevated NCP to any factor or combination of factors. Nevertheless, the associations revealed by our survey characterize traits of high N_2_ fixation + NCP sites in this region, providing a starting point for more targeted investigation of the cause(s) responsible for observed trends.

Similar to prokaryotes, N_2_ fixation and productivity-associated eukaryotic taxa also tend to exhibit strong positive associations with pCu, dCu, pMn, dMn, pCo, pAl, and Silica (Fig. S7) and negative associations with temperature and mixed layer depth (Fig. 6).

Despite the prevalent view that much of the surface ocean is N or Fe-limited, few to no eukaryotic or prokaryotic taxa show associations with nitrate + nitrite, N:P, or dFe (Fig. S7). Nitrate concentrations were typically replete along the coastal section of the survey^1^. Raw inorganic nutrient concentrations also do not necessarily reflect true biological nutrient limitation, but rather the balance between sources and sinks including biological uptake and release. Nutrient ratios can be more informative when attempting to relate community composition to nutrient states^12^. However, we also do not observe significant relationships between the N:P ratio and community data. These results suggest that specific taxonomic abundances across our study region do not generally vary tightly with nitrogen or iron availability.

**Supplementary Methods**

**Potential impact of vertical fluxes upon estimates of net community production**

In addition to biological production and respiration, O_2_/Ar in the surface ocean can also be influenced by several other fluxes. Integrated biological oxygen in the mixed layer can be expressed using the following mass balance:

$$(2) MLD\frac{dO_{2_{bio}}}{dt}=NCP-F_{g}+F_{v}+F_{e}$$

where $O_{2_{bio}}$ denotes the biological oxygen concentration within the mixed layer, F_g_ the gas exchange term, and F_v_ and F_e_ the fluxes resulting from eddy vertical diffusive mixing and from entrainment of subsurface waters into the mixed layer, respectively. We assume that the system is at steady state, with F_g_ equal to NCP, and that vertical and horizontal fluxes of O_2_/Ar are negligible.

To determine the potential effect of F_v_ and F_e_ upon calculated productivity rates, we adopted the approach of Castro-Morales et al., 2013^13^, estimating eddy diffusive fluxes using equation (6) and entrainment fluxes from equation (5). For the study region, we use a vertical eddy diffusivity coefficient of 1 x 10^-5^ m^2^ s^-1^, taken from Wallace et al., 1994^14^. Oxygen gradients were calculated from CTD profile data using a Model-II bisector least-squares fit. Mixed-layer depths prior to the date of measurement were taken from the JAMSTEC Argo Grid Point Value MLD dataset^15^. As Argo MLD data coverage for the study region was poor, we also conducted a sensitivity analysis by assuming, at each CTD location, a prior 3 m entrainment over the median calculated residence time of a given year’s cruise. Estimated depth-integrated F_v_ fluxes were low, ranging between -2.9 and 0.6 mmol O_2_ m^-2^ d^-1^ (range of integrated NCP values for all three years was -24.5 to 163.4 mmol O_2_ m^-2^ d^-1^). Sparse Argo MLD data availability for the study region allowed only a few estimates of entrainment flux to be made (n=6), although calculated fluxes were also generally low (0 to -5.5 mmol O_2_ m^-2^ d^-1^). Assuming a 3 m entrainment at each CTD location over the course of the median residence time of oxygen during each cruise produced entrainment fluxes ranging between -6.5 and 1.4 mmol O_2_ m^-2^ d^-1^.

These estimates also likely represent an upper bound to the influence of vertical fluxes. The open-ocean Sargasso Sea is seasonally stratified, meaning that entrainment fluxes in the subtropical area of the study region are likely limited. We observed in all three years that total oxygen concentrations generally increased with depth, meaning that any vertical fluxes from the subsurface would increase measured mixed-layer NCP. Consequently, negative NCP values in the open ocean are likely to result from true net heterotrophy as opposed to vertical mixing. Additionally, much of the variability in O_2_ saturation with depth is likely thermally-driven. At the same time, subsurface maxima in O_2_/Ar are common in the subtropical oceans during summer and typically result from accumulation of biological oxygen below the stratified thermocline^16,17^. Consequently, part of this increase in O_2_ below the mixed layer in the open ocean is likely attributable to biology. The O_2_/Ar method remains more limited in coastal waters, however, where vertical advection and other mixing may be more dynamic. On and near the coastal shelf, our calculations are unable to account for such factors, and low/negative NCP rates should be interpreted with greater caution. Ultimately, only five molecular stations exhibited undersaturation greater than the error inherent to the O_2_/Ar method (ΔO_2_/Ar + / - 0.3%), so these measurements have negligible effects on our productivity-taxonomy analyses.

**Nutrient and trace metal sampling**

Surface seawater for nutrient analyses (NO_2_/NO_3_, PO_4_, and silicate) were collected into 15 mL Falcon tubes, sealed, and preserved at -20°C immediately (silicate samples were preserved at 4°C). Concentrations were subsequently analyzed using an Automatic Nutrients Analyzer. The detection limits of the method used were 0.03 µM for NO_2_+NO_3_, 0.014 µM for PO_4_, and 0.05 µM for silicate.

Sample seawater for O_2_/Ar, N_2_ fixation, and trace metals was pumped aboard ship using a trace-metal clean towfish (Geofish) and Teflon plumbing^18^. For dissolved trace metal measurements, seawater was pumped continuously from the towfish into a trace metal clean environment (double-layered plastic bubble) pressurised by air from a class-100 laminar flow hood for sampling. The cleaning protocols for sampling bottles and equipment followed GEOTRACES guidelines^19^. Water taken for analysis of dissolved trace metals was filtered through a 0.2 μm capsule filter (Sartorius SARTOBRAN® 300). Seawater was collected in acid-cleaned 60 mL LDPE bottles, after rinsing three times with about 20 mL of seawater. Samples were then acidified to ~pH 1.7 with 2 ‰ (v/v) HCl (Ultrapur® Merck) in the laminar flow hood. The sample bottles were then double bagged and stored at ambient temperature in the dark before analysis on shore. All trace metal sampling as well as macronutrient sampling was conducted at timings precisely coinciding with the collection of samples for DNA extraction.

Particulate trace elements were collected on polyethersulfone filters (0.45 μm supor®) mounted in Nalgene polycarbonate filtration units. Filters were cleaned according to Planquette et al., 2012^20^ and stored in Milli-Q in acid-cleaned, low density polyethylene bottles (Nalgene) until use. Filtration units were soaked in 0.1 M HCl (suprapur grade, Merck) and rinsed with ultrapure water (Milli-Q, 18 MΩ.cm), between each use. Seawater was collected in 2 L LDPE bottles then poured into the filtration unit until the filter was clogged, and the volume recorded. Using plastic tweezers rinsed with Milli-Q between samples, filters were then transferred to acid-cleaned polystyrene Petri slides (Millipore), double bagged and stored frozen at -20**°**C until analysis. Both dissolved (dTM) and particulate (pTM) fractions of trace metals were determined by SF-ICP-MS.

**Trace metal analytical procedures**

*Dissolved trace metals*

Seawater samples were analyzed following the protocol of Lagerström et al., 2013^21^. Briefly, samples were introduced to a PFA-ST nebulizer and a cyclonic spray chamber via a SeaFAST-picoTM introduction system (Elemental Scientific Incorporated, Omaha, NE). A six-point calibration curve was prepared by standard additions of a mixed element standard to our in-house standard seawater (North Atlantic seawater collected during GA01 cruise, see Supplementary Table 2), which was run at the beginning, the middle and the end of each run. Every 10th sample, a replicate was run, and accuracy was determined from analysis of consensus (SAFe S, GSP) and certified (NASS-7) seawaters at the beginning, middle and end of each run.

*Particulate trace elements*

Sample handling was performed in a Class-100 clean room back in the home laboratory. Particulate samples were digested following the protocol described by Planquette et al., 2012^20^. Briefly, filters were placed on the inner wall of acid-clean 15 mL PFA vials (Savillex), and 2 mL of a solution containing 2.9 M hydrofluoric acid (HF, suprapur grade, Merck) and 8 M nitric acid (HNO_3_, Ultrapur grade, Merck) was added to each vial. Vials were then sealed and refluxed at 130°C on a hot plate for 4 h. Once cool, the digest solution was evaporated until near-dryness. Subsequently, 100 µL of concentrated HNO_3_ (Ultrapur grade, Merck) was added, and the solution was re-evaporated to remove fluorides. Finally, 3 mL of 0.8M HNO_3_ (Ultrapur grade, Merck) was added and the archive solution was transferred to an acid cleaned 15 mL polypropylene centrifuge tube (Corning®) and stored at 4°C until analyses.

All analyses were performed on a SF-ICP-MS Element2 (Thermo) at the Pôle Spectrométrie Océans. The archive solution (400 µL) was diluted in 96-well titre plate (ESI) by addition of 2 mL of 0.8 M HNO_3_ (Ultrapur, Merck) spiked with 1 ppb ^115^In, in order to monitor instrument drift. Samples were introduced with a PFA-ST nebulizer connected to a quartz cyclonic spray chamber (Elemental Scientific Incorporated, Omaha, NE) via a modified SC-Fast introduction system consisting of an SC-2 autosampler, a six-port valve and a vacuum rinsing pump. The autosampler was contained under a HEPA filtered unit (Elemental Scientific). Two 6-point, multi-element standard curves with acid matrix identical to that of samples and concentrations bracketing the range of the samples were run at the beginning, the middle and the end of the run. Every 10th sample was a replicate to monitor analytical precision and instrument drift was monitored with ^115^In spiked in all samples. Accuracy was determined by performing digestions of the certified reference material BCR-414 (plankton, Community Bureau of Reference, Commission of the European Communities) following the same protocol as for samples. Recoveries were typically within 10% of the certified values (and within the error of the data, taken from replicate measurements).

**Bioinformatic processing of molecular data**

Illumina output from our 2016+2017 samples consisted of 22 807 458 paired-end reads, which were trimmed, merged, and quality filtered using pandaseq^22^. Mean 18S amplicon length was 424.2 + / - 4.3 bp (mean + / - standard deviation), and mean 16S amplicon length was 296.5 + / - 3.0 bp. Reads were subsequently demultiplexed in QIIME^23^ using the script split_libraries_fastq.py. Chimeric sequences were detected and filtered using the Usearch 6.1 algorithm^24,25^ at 97% similarity using the SILVA 123.1 ribosomal rRNA database^26^. Primer and non-biological sequences were trimmed and filtered using Tagcleaner^27^.

Pandaseq-assembled 2015 sequencing data was processed following the same demultiplexing, chimera detection, and Tagcleaner steps. Open-reference OTU picking was then performed upon 2016+2017 sequences and 2015 sequencing in iterative mode due to memory limitations. A 97% similarity threshold was employed, and OTU picking was performed using the Usearch 6.1 algorithm. The PyNAST^28^ alignment method was employed, and taxonomy assignment was performed using the RDP classifier 2.2^29^. Following OTU picking and taxonomy assignment, internal standard sequences as well as metazoan and chloroplast + mitochondrial sequences were filtered from the dataset.

**Statistical analyses**

Three 16S samples from 2017 to which <5 reads were assigned were excluded from our dataset. One 16S and five 18S samples from 2015 as well as one 16S sample from 2017 were not assigned any reads during demultiplexing. The five defective 18S samples from 2015 were likely the consequence of a faulty forward primer/adapter.

In addition, a number of samples were excluded from quantitative taxonomic analyses on the basis that their internal standard recovery ratios or standard-derived total rDNA gene abundances were statistical outliers. Box-and-whisker plots of log-scaled recovery ratios were used to identify three samples with anomalously-high standard recovery rates relative to other samples of the same type collected in the same year (Fig. S4). One 2017 16S sample was also filtered from the dataset on account of having a total 16S rDNA gene abundance well above two standard deviations from the mean for the 2017 16S dataset, giving it a heavily disproportionate weight in PLS regression analysis and in co-occurrence network module selection. These four filtered samples were not, however, excluded from relative abundance-based analyses such as alpha diversity calculations.

Exclusion of these samples along with samples from depth left 79 18S samples and 79 16S samples in total. Underway metadata was time-averaged around each molecular sampling site using identical criteria as Wang et al., 2018^30^.

For alpha diversity analyses only, 18S and 16S samples were rarefied at an even depth corresponding to the smallest included 18S and 16S library size.

To visualize differences in whole-community structure across samples, principal Coordinates Analysis (PCoA) was performed in the ‘phyloseq’ package^31^ on both the 18S and 16S community datasets, with ecological distances between samples calculated using weighted Bray-Curtis dissimilarities. Samples were color-coded according to whether they were collected at locations north or south of the northern wall of the Gulf Stream in that year. The northern boundary of the Gulf Stream was determined from monthly EN4 temperature data and was defined as the intersection of the 15°C isotherm with the 200 m isobath—a criterion long-accepted as having a high ability to track the northern limit of the Gulf Stream^32,33^.

**WGCNA module selection and network analyses using Flashweave, L-GRAAL**

Eukaryotic and prokaryotic taxa were agglomerated at the genus level (6th taxonomic rank) in phyloseq, then combined into a single abundance table following the removal of samples containing prokaryotic data but no eukaryotic data and vice versa. We forego the Hellinger transformation step, as our abundance data is quantitative. To optimize the scale-free topology fit, we selected a soft-thresholding power of 8, yielding nine ‘modules’ post-merging containing between 31 and 124 genera. Calculation of topological overlap measures followed by hierarchical clustering was executed following the same steps as Guidi et al., 2016^34^. Partial least squares regression analysis to assess the ability of selected modules to predict NCP and N_2_ fixation were then performed following the procedure used in Guidi et al., 2016^34^. For input to Flashweave^35^ and L-GRAAL^36^, we selected the two sub-networks most strongly related to productivity and nitrogen fixation (Pearson = 0.61 and 0.56 for NCP, Pearson = 0.88 and 0.87 for N_2_ fixation, p<<0.001 for both), as well as the module most negatively-associated with NCP (Pearson = -0.12, p>0.05) and N_2_ fixation (Pearson = -0.2, p>0.05) (Fig. S3). Flashweave was run with default parameters (sensitive=TRUE, heterogeneous=FALSE). An alpha value of 0.4 was selected for L-GRAAL alignments as in all pairs of modules it showed a high consensus between sequence and topological similarity while conserving high alignment quality scores (Edge correctness and Symmetric substructure scores). Centrality metrics characterize node importance in a network, with different measures spanning from local to global scales. The generalized keystone index introduced in Estrada et al., 2007^37^ uses a factor analysis approach that reduces dimensionality by transforming a set of centralities in a linear combination, allowing easy ranking of nodes with a single metric. Two local centralities (degree and betweenness centralities), a “meso-scale” centrality (subgraph centrality) and a global centrality (closeness centrality) were considered. HiveAlign (<https://gitlab.univ-nantes.fr/erwan.delage/HiveAlign.git>) was then used to display the produced alignments.

**References:**

1 Tang, W. *et al.* New insights into the distributions of nitrogen fixation and diazotrophs revealed by high-resolution sensing and sampling methods. *The ISME Journal*, doi:10.1038/s41396-020-0703-6 (2020).

2 Tang, W. *et al.* Revisiting the distribution of oceanic N2 fixation and estimating diazotrophic contribution to marine production. *Nat Commun* **10**, 831, doi:10.1038/s41467-019-08640-0 (2019).

3 Brand, L. E., Sunda, W. G. & Guillard, R. R. L. Reduction of marine phytoplankton reproduction rates by copper and cadmium. *Journal of Experimental Marine Biology and Ecology* **96**, 225-250, doi:<https://doi.org/10.1016/0022-0981(86)90205-4> (1986).

4 Wurtsbaugh, W. A. & Horne, A. J. Effects of Copper on Nitrogen Fixation and Growth of Blue-Green Algae in Natural Plankton Associations. *Canadian Journal of Fisheries and Aquatic Sciences* **39**, 1636-1641, doi:10.1139/f82-220 (1982).

5 Levy, J. L., Stauber, J. L. & Jolley, D. F. Sensitivity of marine microalgae to copper: The effect of biotic factors on copper adsorption and toxicity. *Science of The Total Environment* **387**, 141-154, doi:<https://doi.org/10.1016/j.scitotenv.2007.07.016> (2007).

6 Maldonado, M. T., Hughes, M. P., Rue, E. L. & Wells, M. L. The effect of Fe and Cu on growth and domoic acid production by Pseudo-nitzschia multiseries and Pseudo-nitzschia australis. *Limnology and Oceanography* **47**, 515-526, doi:10.4319/lo.2002.47.2.0515 (2002).

7 Moeller, P. D. R. *et al.* Metal Complexes and Free Radical Toxins Produced by Pfiesteria piscicida. *Environmental Science & Technology* **41**, 1166-1172, doi:10.1021/es0617993 (2007).

8 Long, M. *et al.* A multi-traits approach reveals the effects of Cu on the physiology of an allelochemical-producing strain of Alexandrium minutum. (Submitted).

9 Bruland, K. W., Middag, R. & Lohan, M. C. in *Treatise on Geochemistry, 2nd Edition* (eds H.D. Holland & K.K. Turekian) Ch. 8.2, 19-51 (Elsevier, 2014).

10 Mann, E. L., Ahlgren, N., Moffett, J. W. & Chisholm, S. W. Copper toxicity and cyanobacteria ecology in the Sargasso Sea. *Limnology and Oceanography* **47**, 976-988, doi:10.4319/lo.2002.47.4.0976 (2002).

11 Orians, K. J. & Bruland, K. W. The biogeochemistry of aluminum in the Pacific Ocean. *Earth and Planetary Science Letters* **78**, 397-410, doi:<https://doi.org/10.1016/0012-821X(86)90006-3> (1986).

12 Mousing, E., Richardson, K. & Ellegaard, M. Global patterns in phytoplankton biomass and community size structure in relation to macronutrients in the open ocean. *Limnology and Oceanography* **0**, doi:10.1002/lno.10772 (2018).

13 Castro-Morales, K., Cassar, N., Shoosmith, D. R. & Kaiser, J. Biological production in the Bellingshausen Sea from oxygen-to-argon ratios and oxygen triple isotopes. *Biogeosciences* **10**, 2273-2291, doi:10.5194/bg-10-2273-2013 (2013).

14 Wallace, D. W. R. ANTHROPOGENIC CHLOROFLUOROMETHANES AND SEASONAL MIXING RATES IN THE MIDDLE ATLANTIC BIGHT. *Deep-Sea Res Pt Ii* **41**, 307-324, doi:10.1016/0967-0645(94)90025-6 (1994).

15 Hosoda, S., Ohira, T., Sato, K. & Suga, T. Improved description of global mixed-layer depth using Argo profiling floats. *Journal of Oceanography* **66**, 773-787, doi:10.1007/s10872-010-0063-3 (2010).

16 Spitzer, W. S. & Jenkins, W. J. RATES OF VERTICAL MIXING, GAS-EXCHANGE AND NEW PRODUCTION - ESTIMATES FROM SEASONAL GAS CYCLES IN THE UPPER OCEAN NEAR BERMUDA. *Journal of Marine Research* **47**, 169-196, doi:10.1357/002224089785076370 (1989).

17 Hamme, R. C. & Emerson, S. R. Constraining bubble dynamics and mixing with dissolved gases: Implications for productivity measurements by oxygen mass balance. *Journal of Marine Research* **64**, 73-95, doi:10.1357/002224006776412322 (2006).

18 Bruland, K. W., Rue, E. L., Smith, G. J. & DiTullio, G. R. Iron, macronutrients and diatom blooms in the Peru upwelling regime: brown and blue waters of Peru. *Marine Chemistry* **93**, 81-103, doi:<https://doi.org/10.1016/j.marchem.2004.06.011> (2005).

19 Cutter, G. *et al.* (GEOTRACES, 2010).

20 Planquette, H. & Sherrell, R. M. Sampling for particulate trace element determination using water sampling bottles: methodology and comparison to in situ pumps. *Limnology and Oceanography: Methods* **10**, 367-388, doi:doi:10.4319/lom.2012.10.367 (2012).

21 Lagerström, M. E. *et al.* Automated on-line flow-injection ICP-MS determination of trace metals (Mn, Fe, Co, Ni, Cu and Zn) in open ocean seawater: Application to the GEOTRACES program. *Marine Chemistry* **155**, 71-80, doi:<https://doi.org/10.1016/j.marchem.2013.06.001> (2013).

22 Masella, A. P., Bartram, A. K., Truszkowski, J. M., Brown, D. G. & Neufeld, J. D. PANDAseq: PAired-eND Assembler for Illumina sequences. *Bmc Bioinformatics* **13**, doi:10.1186/1471-2105-13-31 (2012).

23 Caporaso, J. G. *et al.* QIIME allows analysis of high-throughput community sequencing data. *Nature Methods* **7**, 335-336, doi:10.1038/nmeth.f.303 (2010).

24 Edgar, R. C. Search and clustering orders of magnitude faster than BLAST. *Bioinformatics* **26**, 2460-2461, doi:10.1093/bioinformatics/btq461 (2010).

25 Edgar, R. C., Haas, B. J., Clemente, J. C., Quince, C. & Knight, R. UCHIME improves sensitivity and speed of chimera detection. *Bioinformatics* **27**, 2194-2200, doi:10.1093/bioinformatics/btr381 (2011).

26 Pruesse, E. *et al.* SILVA: a comprehensive online resource for quality checked and aligned ribosomal RNA sequence data compatible with ARB. *Nucleic Acids Research* **35**, 7188-7196, doi:10.1093/nar/gkm864 (2007).

27 Schmieder, R., Lim, Y. W., Rohwer, F. & Edwards, R. TagCleaner: Identification and removal of tag sequences from genomic and metagenomic datasets. *Bmc Bioinformatics* **11**, doi:10.1186/1471-2105-11-341 (2010).

28 Caporaso, J. G. *et al.* PyNAST: a flexible tool for aligning sequences to a template alignment. *Bioinformatics* **26**, 266-267, doi:10.1093/bioinformatics/btp636 (2010).

29 Wang, Q., Garrity, G. M., Tiedje, J. M. & Cole, J. R. Naive Bayesian classifier for rapid assignment of rRNA sequences into the new bacterial taxonomy. *Applied and Environmental Microbiology* **73**, 5261-5267, doi:10.1128/aem.00062-07 (2007).

30 Wang, S., Lin, Y., Gifford, S., Eveleth, R. & Cassar, N. Linking patterns of net community production and marine microbial community structure in the western North Atlantic. *The ISME Journal*, doi:10.1038/s41396-018-0163-4 (2018).

31 McMurdie, P. J. & Holmes, S. phyloseq: An R Package for Reproducible Interactive Analysis and Graphics of Microbiome Census Data. *Plos One* **8**, doi:10.1371/journal.pone.0061217 (2013).

32 Joyce, T. M., Deser, C. & Spall, M. A. The relation between decadal variability of subtropical mode water and the North Atlantic Oscillation. *Journal of Climate* **13**, 2550-2569, doi:10.1175/1520-0442(2000)013<2550:trbdvo>2.0.co;2 (2000).

33 Fuglister, F. C. Alternative analyses of current surveys. *Deep Sea Research (1953)* **2**, 213-229, doi:<https://doi.org/10.1016/0146-6313(55)90026-5> (1955).

34 Guidi, L. *et al.* Plankton networks driving carbon export in the oligotrophic ocean. *Nature* **532**, 465-+, doi:10.1038/nature16942 (2016).

35 Tackmann, J., Rodrigues, J. F. M. & von Mering, C. Rapid inference of direct interactions in large-scale ecological networks from heterogeneous microbial sequencing data. *bioRxiv* (2018).

36 Malod-Dognin, N. & Pržulj, N. L-GRAAL: Lagrangian graphlet-based network aligner. *Bioinformatics* **31**, 2182-2189, doi:10.1093/bioinformatics/btv130 (2015).

37 Estrada, E. Characterization of topological keystone species: Local, global and “meso-scale” centralities in food webs. *Ecological Complexity* **4**, 48-57, doi:<https://doi.org/10.1016/j.ecocom.2007.02.018> (2007).
